# Diversifying history: A large-scale analysis of changes in researcher demographics and scholarly agendas

**DOI:** 10.1371/journal.pone.0262027

**Published:** 2022-01-19

**Authors:** Stephan Risi, Mathias W. Nielsen, Emma Kerr, Emer Brady, Lanu Kim, Daniel A. McFarland, Dan Jurafsky, James Zou, Londa Schiebinger

**Affiliations:** 1 Program in Digital Humanities, Massachusetts Institute of Technology, Cambridge, Massachusetts, United States of America; 2 Department of Sociology, University of Copenhagen, Copenhagen, Denmark; 3 Stanford Graduate School of Education, Stanford University, Stanford, California, United States of America; 4 Department of Political Science, Aarhus University, Aarhus, Denmark; 5 Department of Linguistics, Department of Computer Science, Stanford University, Stanford, California, United States of America; 6 Department of Biomedical Data Science, Stanford University, Stanford, California, United States of America; 7 Department of History, Stanford University, Stanford, California, United States of America; University of California San Francisco, UNITED STATES

## Abstract

**Background:**

In recent years, interest has grown in whether and to what extent demographic diversity sparks discovery and innovation in research. At the same time, topic modeling has been employed to discover differences in what women and men write about. This study engages these two strands of scholarship to explore associations between changing researcher demographics and research questions asked in the discipline of history. Specifically, we analyze developments in history as women entered the field.

**Methods:**

We focus on author gender in diachronic analysis of history dissertations from 1980 (when online data is first available) to 2015 and a select set of general history journals from 1950 to 2015. We use correlated topic modeling and network visualizations to map developments in research agendas over time and to examine how women and men have contributed to these developments.

**Results:**

Our summary snapshot of aggregate interests of women and men for the period 1950 to 2015 identifies new topics associated with women authors: gender and women’s history, body history, family and households, consumption and consumerism, and sexuality. Diachronic analysis demonstrates that while women pioneered topics such as gender and women’s history or the history of sexuality, these topics broaden over time to become methodological frameworks that historians widely embraced and that changed in interesting ways as men engaged with them. Our analysis of history dissertations surface correlations between advisor/advisee gender pairings and choice of dissertation topic.

**Conclusions:**

Overall, this quantitative longitudinal study suggests that the growth in women historians has coincided with the broadening of research agendas and an increased sensitivity to new topics and methodologies in the field.

## Introduction

In recent years, there has been much talk of demographic diversity sparking discovery and innovation [1–3]. The questions are: When outsiders enter an academic field, do they create new knowledge? Do newcomers launch new areas of research? These questions revive a longstanding discussion in science studies concerning the historical relationship between who creates knowledge and the knowledge produced [4, 5].

We add to this discussion by exploring associations between changing researcher demographics and research questions asked in the field of history. Specifically, we analyze developments in the discipline of history as women entered the field. We focus on gender in diachronic analysis of history dissertations from 1980 (when online data is first available) to 2015 and a select set of general history journals from 1950 to 2015. We use correlated topic modeling [6] and network visualizations to map developments in research agendas over time and to examine how women and men have contributed to these developments.

A flurry of articles has emerged in the past decade that employ topic modeling to discover differences in what women and men write about. In political science, women tend to focus on gender, race and ethnicity, interest groups, health-care, while men tend to engage with theory, statistics, voting, campaigns, and interstate war [7, 8]. Findings reveal similar patterns in sociology, economics, management, and medicine [9–12]. In some cases, women are identified as bringing new and innovative perspectives, but those contributions tend to be devalued in predominantly male fields, such as the natural sciences [13].

This quantitative, longitudinal study engages these various strands of scholarship to explore associations between changing researcher demographics and research questions asked in the discipline of history. First, we provide a summary snapshot of aggregate interests of women and men for the period 1950 to 2015 and identify the new topics associated with women authors: gender and women’s history, body history, family and households, consumption and consumerism, and sexuality. We then move into patterns of change over time. While our analysis demonstrates that women pioneered women’s and gender history and the history of sexuality, for example, these topics broaden over time to become methodological frameworks that historians widely embraced and that changed in interesting ways as scholars of all genders engaged with them. We then pivot to training the next generation and surface correlations between advisor/advisee gender pairings and choice of dissertation topic. Overall, this study suggests that the growth in women historians has coincided with the broadening of research agendas and an increased sensitivity to new topics and methodologies in the field.

## Materials and methods

The first dataset consists of 10,367 full-text articles for the period 1951 to 2014 provided by JSTOR from a set of History journals published in the US. These include the American Historical Review, Journal of American History (previously the Mississippi Valley Historical Review), Journal of Modern History, Journal of Social History, Journal of World History, History and Theory, Journal of Interdisciplinary History, Ethnohistory, Comparative Studies in Society and History. Each article in the dataset includes a title, full text, the author’s first and last names, and year of publication.

**Fig 1:**
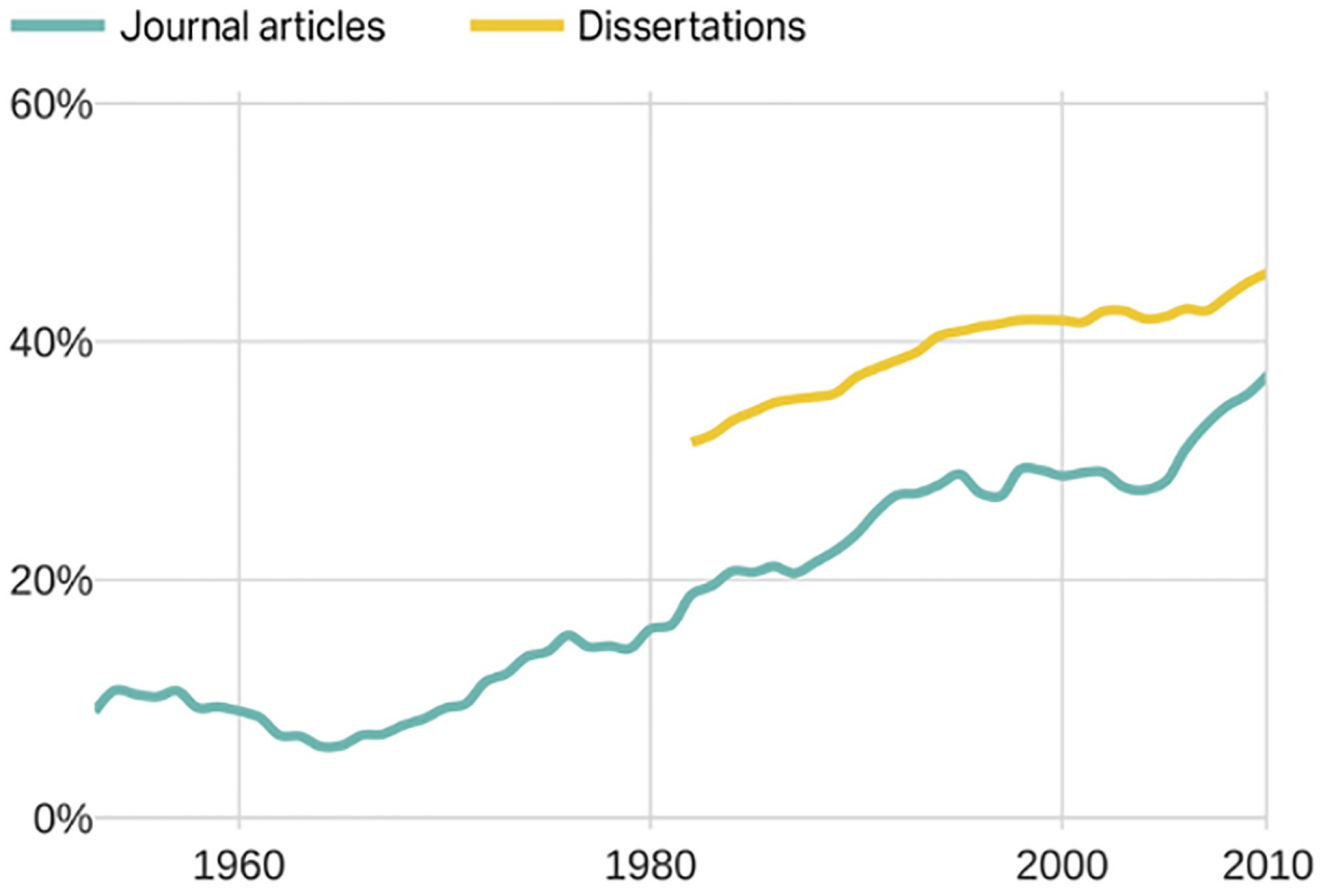
Share of history journal articles and dissertations written by women. Fig 1 shows the percent of journal articles and dissertations written by women using a five-year moving average to smooth out spikes. In 1982, women wrote 31% of all history dissertations. Women, however, published only 20% of journal articles. Even though women continue on an upward trajectory of dissertation writing, men continue proportionately to publish more articles. In 2010, women accounted for 46% of dissertations but only 37% of journal articles.

A second dataset consists of 21,548 history dissertation abstracts written in the US between 1980 (when dissertations are first digitized) and 2015 extracted from ProQuest Dissertation & Theses. Each entry includes the author’s first and last names, the field, date, institution granting the PhD, and, for 71% of all theses, the name of advisor (Fig 1).

In this article, we used correlated topic modeling (CTM) to generate topics using full-text journal articles. CTM is a natural language processing technique that uncovers latent dimensions, called “topics,” in text corpora. These topics are sets of terms, variably weighted by how frequently they are employed together in texts. Notably, as latent dimensions of weighted terms, some of the same terms are employed in multiple topics—but in different combinations and frequencies with other terms (i.e., polysemy is possible and represents an advantage over clustering methods). We used a variety of fitness metrics to determine 90 topics that best predict the terms employed in the corpus and display the best semantic coherence (Texts A and B in S1 File; S1 Fig).

We assigned a label to each topic based on the most important terms belonging to the topic as well as article titles that scored the highest for each topic. For the topic labeled “women and gender,” for example, the highest scoring words are “women,” “men,” “female,” and “gender,” and the highest scoring article is: “‘To Educate Women into Rebellion’: Elizabeth Cady Stanton and the Creation of a Transatlantic Network of Radical Suffragists” (1994). A single topic, however, can reflect different sub-themes and emphases over time. When we look carefully at article titles included in the topic labeled “sexuality,” for instance, we find that in the 1980s it reflected how historians wrote about Freud and psychohistory, and in the 2000s it reflected the study of sexuality and emotions.

We infer author gender using Social Security Administration data, Python gender-guesser, and, where relevant, hand labeling as described in Text C in S1 File. In order to identify how much women and men contribute to each topic, we calculated the average weight of a topic among men and divided it by the average weight summed over women and men. At a value of 0, this identifies topics exclusively studied by women, and at a value of 1, it identifies topics exclusively discussed by men. As such, the measure reflects the proportion of women’s contribution to topics in history while adjusting for the fact that, within our dataset, men published 3.72 times as many articles as women.

Our work has three limitations and ethical concerns important to historians. First, current name algorithmic tools do not provide categories beyond the binary “woman” or “man.” People may identify as multiple and/or non-binary genders [14], and automatically inferring gender of individuals can lead to harm [15–17]. Names are themselves historical artifacts, and naming practices differ within and across cultures, historical eras, and geographic locations. This article uses gender inference as a way of analyzing large-scale dynamics and to investigate how historical knowledge has changed with women (in the aggregate) entering the discipline at higher rates, and not to determine an individual’s gender [18]. We hope future algorithmic tools will allow for large-scale analysis of the role of nonbinary and LGBTQI+ scholars in crafting new fields of enquiry. Historians do this qualitatively, but big data tools have not kept pace.

Second, the existing data and tools cannot classify the authors’ ethnicity and hence do not allow for intersectional approaches. There are several issues here. First, the US population consists of highly mixed ethnicities, and many first names are common across multiple ethnic groups. This means that, when deploying these types of tools, we may identify a name as 25% likely African American and then inappropriately attribute that ethnicity to that individual. Second, sample sizes make quantitative analysis difficult. This is particularly important because 77.8% of all history PhD recipients in 2018 were white, and white women earned 76.1% of all PhDs granted to women [19].

Third, our analysis did not include books (the preferred publication format of most academic historians) because books are not available in a full-text dataset for analysis. Nonetheless, a comparison of trends and patterns across our dissertation and journal samples converge to yield broadly consistent patterns of gender-related topics.

The limitations of the tools mean that our study focuses on the broad categories of women and men and could not investigate patterns in historical research agendas introduced by multiple and/or nonbinary genders; nor could we take intersectional approaches that analyze how the patterns we identify shift as gender, sexual orientation, race/ethnicity and other social categories interact.

## Results

**Fig 2:**
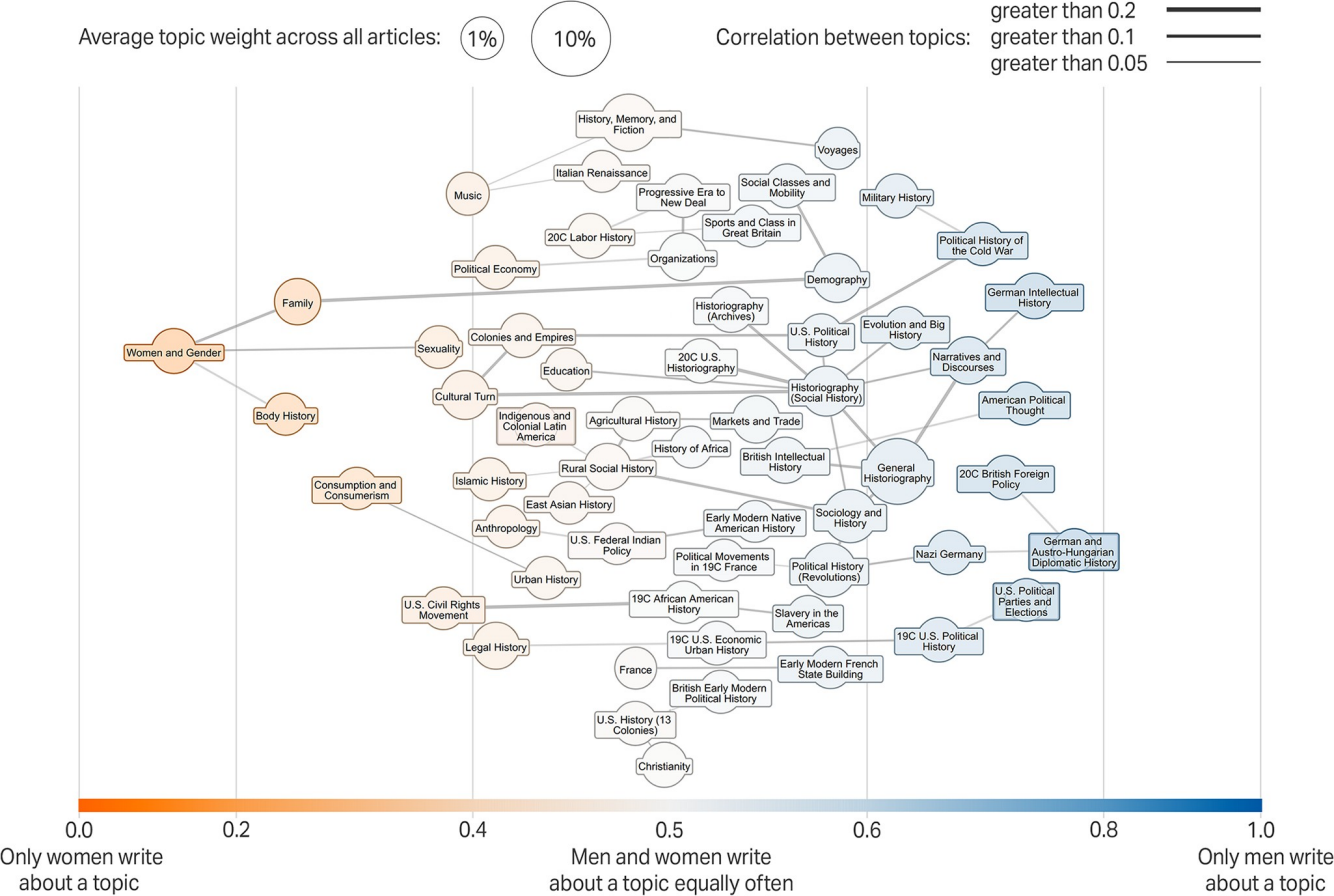
Gender variations in research topics for journal articles, 1951–2014.

Fig 2 provides a snapshot of the topics that women and men treated over the period from 1950 to 2015. In the field of history, men and women explored many of the same topics, including religion, colonialism, African history, and aspects of historiography. Yet, there is notable divergence. Men tended to focus on topics related to political and intellectual history, military history, and Big History (i.e., the study of history on a large scale) [20] Women historians tended to write about topics related to gender, body history, patterns of consumption, family and households, sexuality, the US civil rights movement, and the cultural turn [21].

This image visualizes the 58 of our 90 topics with an average weight greater than 0.7% across all articles. Node sizes indicate the relative prevalence of the topics. The gray connecting lines display associations between topics [22], i.e., their degree of correlation in texts. For each topic, we visualize the strongest connection to another topic. In addition, we include gray connecting lines if two topics were in the top 1% (correlation greater than 0.126) of strongest correlated topics. The y axis has no meaning: top and bottom are for purposes of visualization only; “music” is no more significant than “legal history,” for example.

Women are well-represented among the pioneers of women’s and gender history [23]. As Fig 2 reveals the “women and gender” topic is an outlier with a score of 0.12, which means that the average weight for this topic is eight times higher among women than among men (the women and gender topic on average has a weight of 3.92% among women historians and 0.49% among men historians). Indeed, among the 100 articles with the highest weight for this topic—meaning articles that established the field, its terms, and questions—only seventeen were written by men, and five of those were co-authored with women (Table A in S1 File). While an article is not necessarily influential because it includes a lengthy discussion of women or gender, influential articles often discuss a topic at length to establish its major concepts and questions, as is the case for a number of articles in this dataset.

**Fig 3:**
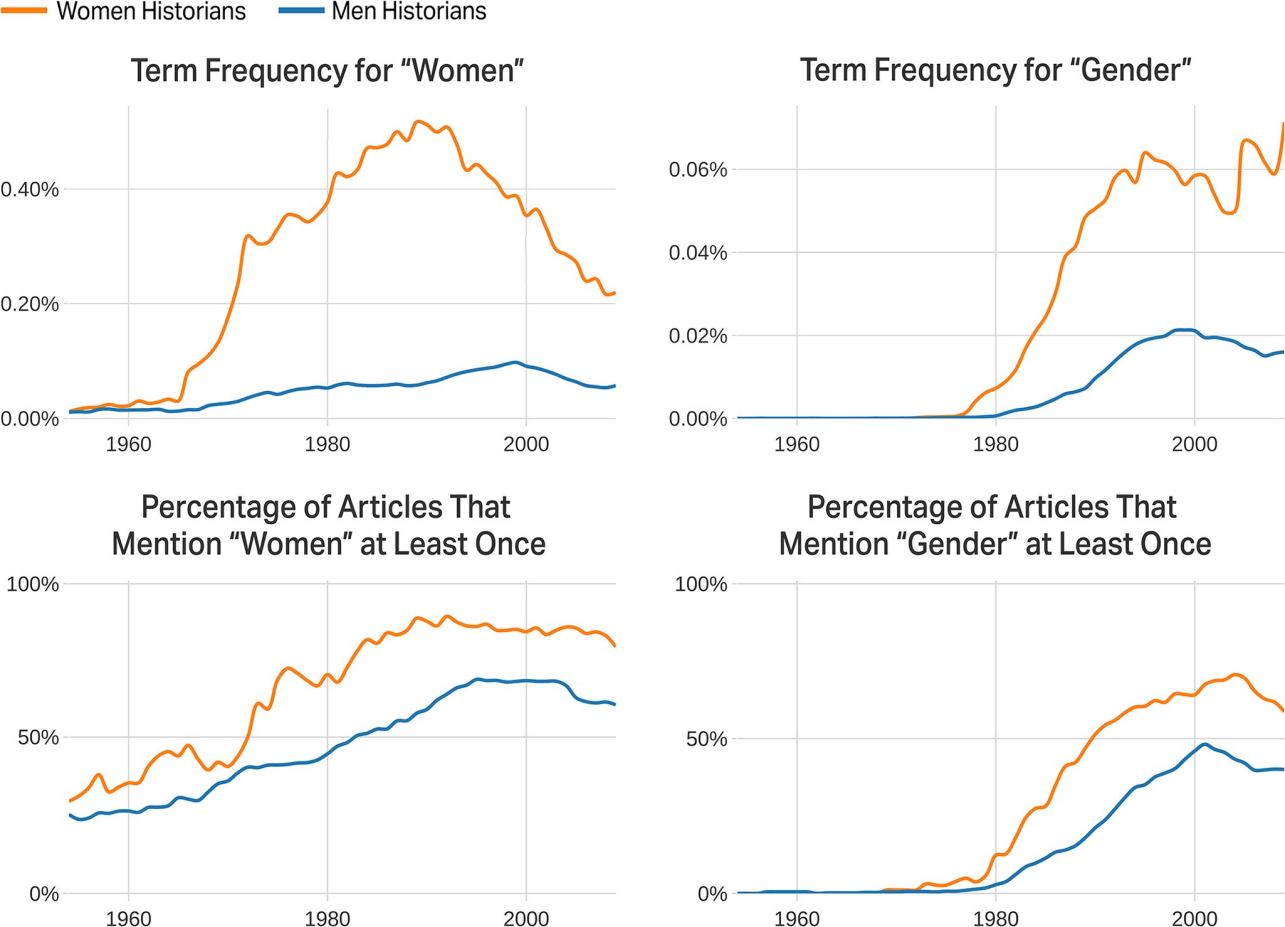
The terms “women” and “gender” in journal articles.

If we look at the frequencies of the two most important terms for the topic “women and gender” (Fig 3), we see that “women” became a focus area of historical research in the US historical profession in the late 1960s, while “gender,” a more complex construct, emerged as an analytical category in the late 1970s.

Fig 3 shows that women pioneered women’s and gender history. About 18% of all articles written by women in the journals dataset score in the top 5% for this topic, meaning that women are more likely to publish on these topics (S2 Fig and Table B in S1 File). Fig 3 shows that men, too, incorporated these methodologically rich and still evolving approaches into their work. Interestingly, men found gender to be a particularly useful analytic, especially when writing the histories of gay men and masculinities. Among articles by men mentioning gender at least 10 times, “gay” and “masculinity” are two of the most distinctive terms (Table C in S1 File).

While some historians have voiced concerns that women’s and gender history has been ghettoized [24], we have found to the contrary that this new methodology has found widespread use among both men and women (Fig 3). Even though “women” as historical subjects are still rarely the focus of articles written by men, we observe an increase in men-authored articles using the term “women” at least once, from 25% in 1954 to 60% in 2009. Readers expect to know what women are doing as historical actors, and this information has been mainstreamed into historical narratives.

**Fig 4:**
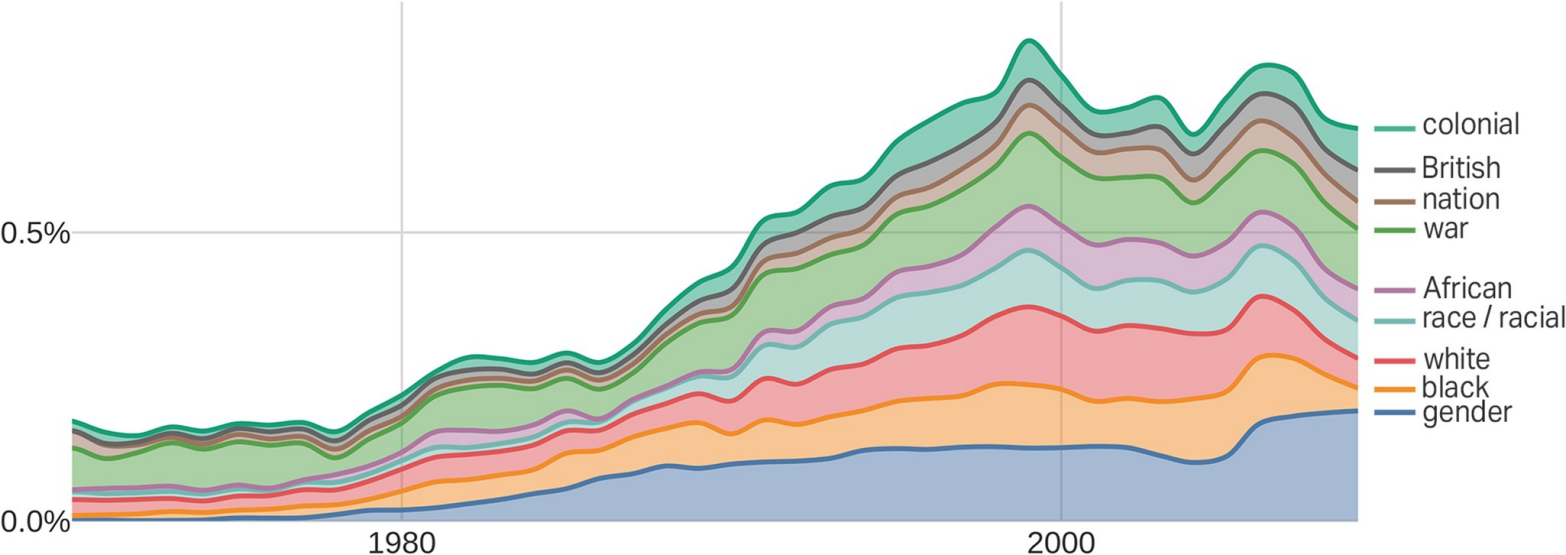
The “women and gender” topic broadened significantly after 1990. This figure shows the stacked term frequencies of the 10 terms most overrepresented in the “women and gender” topic from 1990 to 2009 compared to 1970 to 1989. A stacked display means that the frequencies are displayed one on top of the other. The term frequency of “African,” for example, does not start at the zero line but rather on top of the “race/racial” line.

Fig 4 reveals further that women’s and gender history has broadened over time to become a topic and methodological framework that a majority of historians embraced. It started by conceptualizing mostly white women’s experience in Western culture with topics such as separate spheres, bourgeois feminism, women’s labor history, family and household, and the “other” as political symbol. But after 1990, topics exploded to encompass women, gender, race, and colonialism, with titles such as “From Free Womb to Criminalized Woman: Fertility Control in Brazilian Slavery and Freedom,” “European Nurses and Governesses in Indian Princely Households,” and “Decrying White Peril: Interracial Sex and the Rise of Anticolonial Nationalism in the Gold Coast” (Tables D and E in S1 File). According to the American Historical Association (AHA), women’s and gender history was the topic area in which the share of history specialists increased the most between 1975 and 2015 [25]. By 1995, 80% of History Departments had at least one faculty member specializing in women’s/gender history [26].

**Fig 5:**
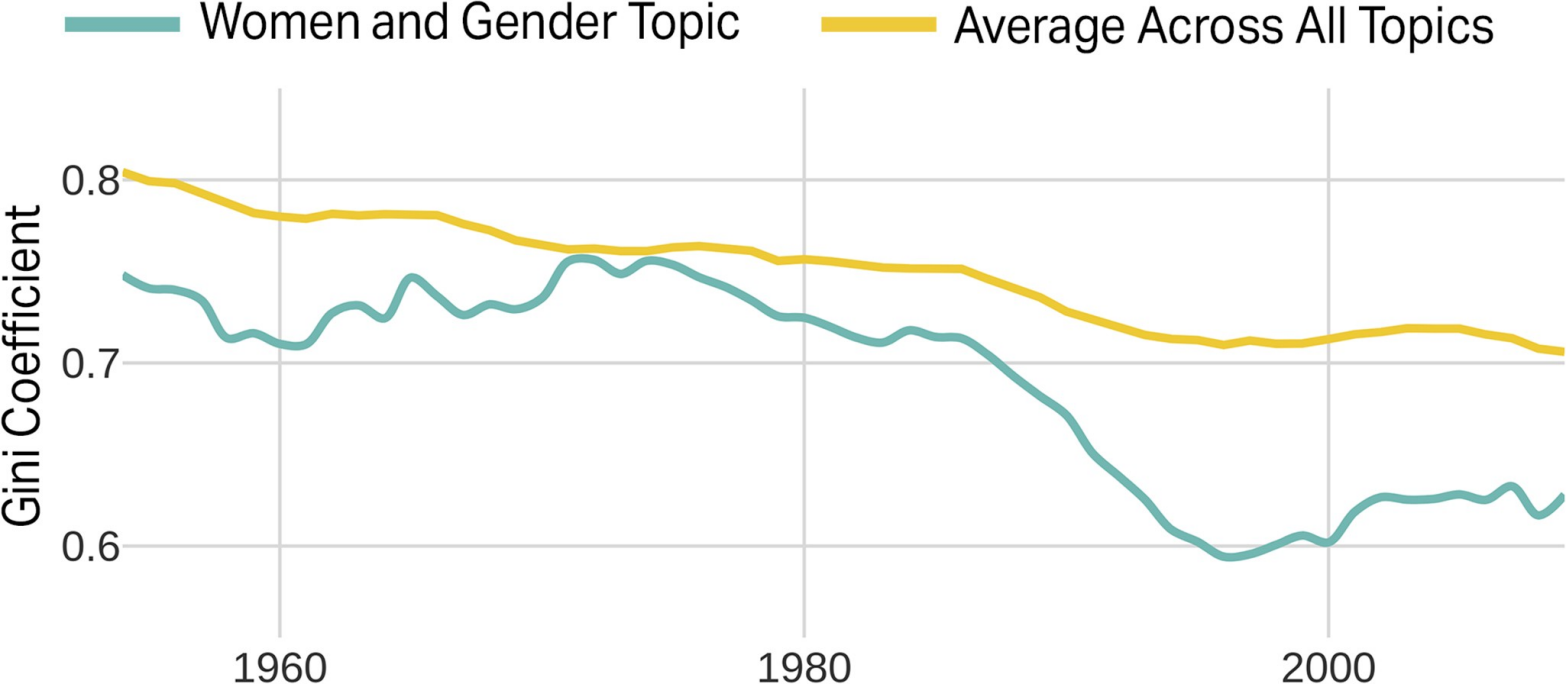
Mainstreaming women and gender into history.

We can also track this shift in our dataset quantitatively by using Gini coefficients as a measure for how frequently the women and gender topic is used in combination with other topics (Fig 5). Here we measure the Gini coefficient for the frequency of topic “co-usage,” meaning when two or more topics appear in the same article. For example, should the women and gender topic appear equally with other topics, then the Gini will be low and approach zero. Should the women and gender topic appear on its own to the exclusion of other topics, the Gini will be high and approach values of one (Text D in S1 File). Our analyses find the women and gender topic consistently has a Gini coefficient comparable to that of other history topics (see average across all topics) from ~1955–1990 but dropped from 0.76 in 1975 to less than 0.6 by the late 1990s. This provides additional evidence that women and gender topics were increasingly adopted in other areas of historical research.

It is important to point out that without the contributions of authors whom our tools identified as men, gender history might be a narrower field. As noted above, within gender history, men took a special interest in gay history and the history of masculinities, with titles such as “Christian Brotherhood or Sexual Perversion? Homosexual Identities and the Construction of Sexual Boundaries in the World War One Era,” “Unacceptable Mannerisms: Gender Anxieties, Homosexual Activism, and Swish in the United States,” “Western Masculinities in War and Peace,” and “Indian Clubs and Colonialism: Hindu Masculinity and Muscular Christianity” (for additional detail, see S1 Data). The same is true, in reverse, for military history, typically a male topic. If we look, for example, at the top ten percent topics that have the heaviest weights for the topic “military history,” we see that 13% of the articles written by women bring distinctive, new interests (for additional information on overrepresented topics, see Text E in S1 File). Women historians emphasize the role of women in wartime as nurses, mothers, police, and sex workers. They highlight women’s role in pacifist and civil rights movements, thereby expanding our view of war beyond battles and politics, and contributing to a broader, cultural history of war. Further, women historians wrote about topics not overtly connected to gender, such as film, memory, and documentation, and race consciousness in the origins of the civil rights movement, thus forging broader discussions of the social forces that shaped collective memory (Text F in S1 File).

Women’s and gender history is not the only topic that emerged across this period. Four other major topics emerged in our analysis: body history, consumption and consumerism, family and household, and sexuality (Fig 2 and S3–S6 Figs). The topic “body history” includes articles on doctors, hospitals, wet nurses, midwives, “gynecological surgeries on insane women,” circumcision in America, hysteria, anorexia nervosa, menstruation, masturbation, and the like, and contributes to larger trends in the history of medicine. The topic, “consumption and consumerism,” as part of the material turn in history [27] includes articles on supermarkets, restaurants, material life, sportswear, cookbooks, beer, cleanliness, bathing, etc. Both women and men discuss these topics, but women dominate the top decile by two to one, and the top one percent of articles by about three to one.

Women also dominate the topic “family and household,” which includes articles on kinship, widowhood, illegitimacy, marriage, orphans, and the like. This topic shows two slightly different trends: family, children, and marriage all peak around 1980, while children and parents peak around 2000 (S7 Fig).

**Fig 6:**
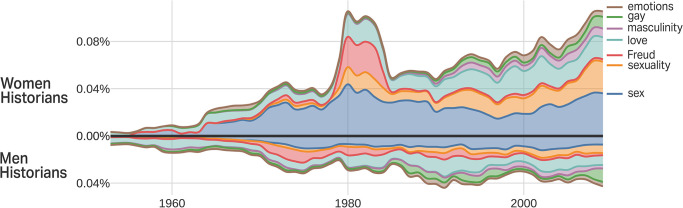
Stacked term frequency chart for “sexuality”.

Finally, the topic “sexuality” is worth further investigation. The analysis in Fig 6 shows the key terms within the historical study of sexuality, which includes sex, love, and Freud. Freud looms large in a first peak in this topic in the late 1970s and early 1980s, when historians both published studies on Freud and tried to develop psychoanalytic methods for doing history. The recent resurgence in this topic (particularly around 2010) extends this prior work and opens to the study of emotion and gay history.

Overall this topic is multifaceted, with the majority of articles on sex, including “The Power of Desire and the Danger of Pleasure: Victorian Sexuality Reconsidered” or “Sexual Foreplay in American Marital Advice Literature.” A number of articles highlight sexuality, including “Sexuality, Race, and Mid-Twentieth Century Social Constructionism” or “Reclaiming the Gay Past.” Some are on love, such as “Romantic Love in the Pre-Modern Period” and “The Pursuit of Married Love,” and some foreground emotions, such as “Transitions in American Emotional Standards for Children” or “Girls, Boys, and Emotions”.

**Fig 7:**
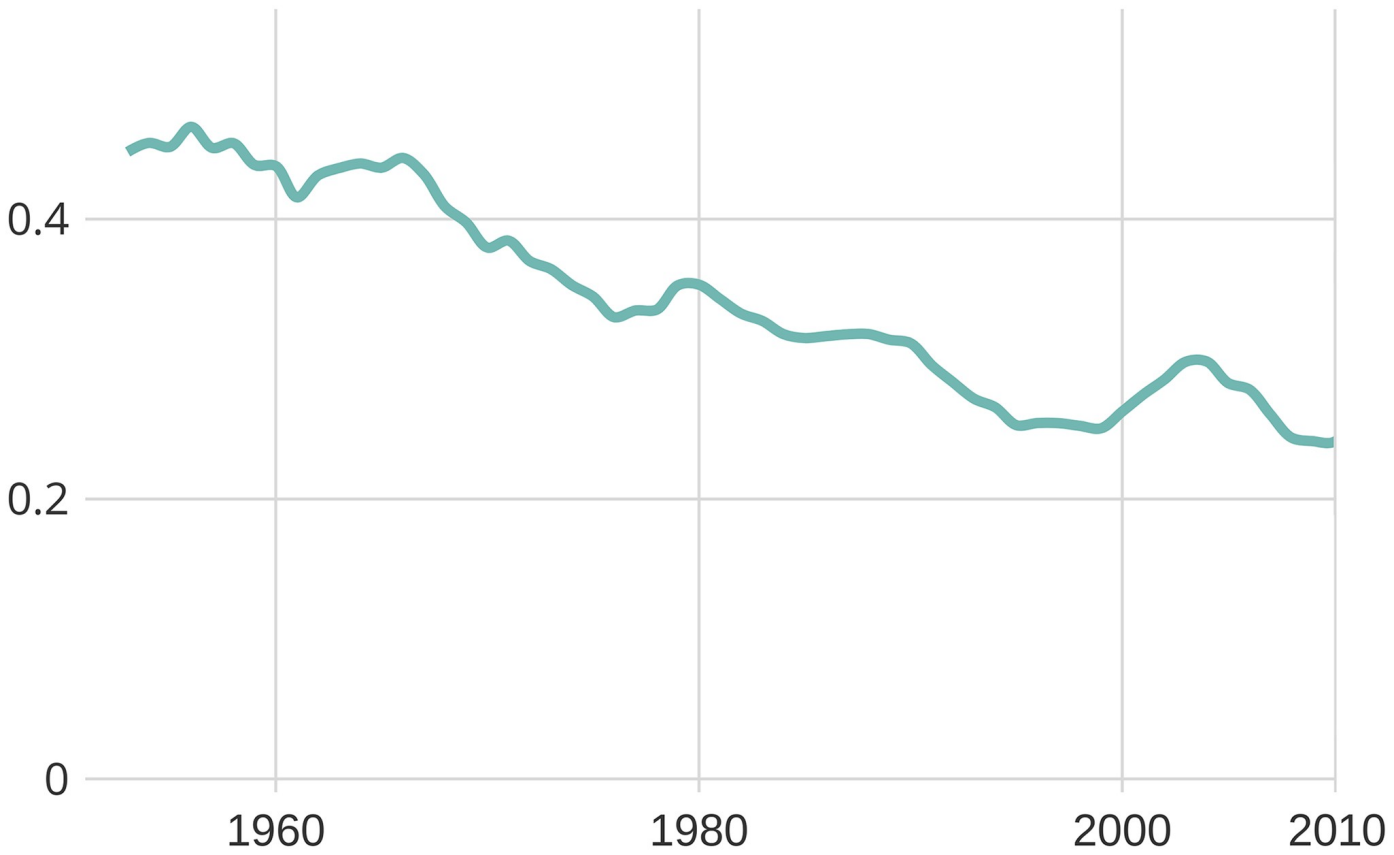
Jensen-Shannon distance between men and women.

A closer inspection of developments in topic similarity suggests that the research interests of women and men have increasingly converged, as the discipline has become more gender integrated. Fig 7 plots the Jensen-Shannon divergence by year—a measure of the distance between women’s and men’s topic distributions—and shows that topic differences were most pronounced in the 1960s, where gender imbalances in representation were stark, and have decreased gradually since then.

Decreasing Jensen-Shannon distance (y-axis) suggests that the research topics between men and women became more similar over time.

### Do advisors who work on gender topics train students who also work on gender topics?

An advisor’s research interests can shape a discipline into the future by the type of students they attract or recruit to the field and the type of advice they offer about choice of dissertation topics [28]. Table 1 reveals that women advisors more often than men attract women students.

**Table 1:** Gender of primary advisor/advisee pairings.

1990s (n = 5260)		Woman Advisor	Man Advisor
	Woman Advisee	11.9%	28.5%
	Man Advisee	7.1%	52.5%
2000s (n = 6303)		Woman Advisor	Man Advisor
	Woman Advisee	16.7%	26.0%
	Man Advisee	11.1%	46.1%

We were able to identify advisors for only about half of our sample. The low quality images of theses early in our dataset made it difficult for optical character recognition to identify advisor names. Further, our data entail information only on primary advisors, not on full committees. We see primary advisors as a proxy for various social interactions we cannot track and observe, and it is a conservative estimate in that the primary advisor plays such an influential role.

We also found a correlation between the advisor/advisee gender pairings and the choice of dissertation topic by analyzing how dissertations from 1990–2020 load on the 90 journal topics discussed above (Text G in S1 File). We do this by assessing how much the document-term matrix for thesis abstracts load on the journal topics—where each journal topic is a weighted vector of terms. In general, topics in the two datasets have a high correspondence.

The top four overrepresented topics for students with a man advisor for dissertations submitted between 1990 and 2015 were: military history, political history of the Cold War, political revolutions, and German and Austro-Hungarian diplomatic history. Those for a woman advisor were: women and gender, consumption and consumerism, the cultural turn, body history, and family (Tables F and G in S1 File).

If we compare weights for the “women and gender” topic in a particular dissertation, we see that having a woman advisor, on average, doubles the weight for this topic compared to having a man advisor (Table 2):

**Table 2:** Weights for the “women and gender” topic.

Woman student/woman advisor	7.7%
Woman student/man advisor	3.9%
Man student/woman advisor	1.4%
Man student/man advisor	0.6%

At the same time, it is important to recognize that women students tend to select gender topics whether or not they study with a woman (woman student/man advisor, 3.9%). Perhaps the most interesting finding here is that women advisors attract men students who either have an interest in gender-related topics or who, through interaction with the advisor, are led to such topics (man student/woman advisor, 1.4%). Clearly, women advisors encourage or advance the topic of women and gender in the academy—both as researchers and as advisors.

Are those students who work on the gender topic successful? Is working on women and gender “professional suicide,” as some PhD candidates were cautioned in the 1980s? As part of this study, we compared researchers who received their PhDs in the 1980s and 1990s and subsequently advised students of their own to those who did not. We found that graduate students who wrote about the cultural turn, labor history, African American history, rural social history, women and gender, slavery and colonialism were more likely than those who wrote on other topics to have students of their own, which suggests that they are employed at a research-intensive institution with a PhD program. The ten most over-represented topics among dissertation writers who subsequently advised students of their own by Dunning’s log-likelihood statistic are: “Cultural Turn,” “20th Century Labor History,” “19th Century African American History,” “Rural Social History,” “Women and Gender,” “Political History (Revolutions),” “Slavery in the Americas,” “Sociology and History,” “Colonies and Empires,” “East Asian History.” (For a further breakdown of dissertation topics among men and women dissertation writers with students (Tables H and I in S1 File). Graduate students who worked on organizations, education, religion, military, or diplomatic history, by contrast, were less likely to have PhD advisees of their own.

## Discussion

Can we attribute the growing popularity of gender history in the 1980s to a “critical mass” of women in the profession? Critical mass theory posits that a “threshold proportion of an underrepresented demographic” is required before a discipline or organization undergoes transformation—in this case, the development of new historical topics and methods [29]. Debate rages about what represents a critical mass. Some scholars posit 10 to 15% of the previously underrepresented group, but most agree that 30% is the point at which change becomes self-sustaining. Empirically, women earned some 28% of PhDs in history in 1981 and 32% by 1985, rising from about 13% in 1970 [30]. By the mid-1980s, predominantly white women had reached critical mass in history PhDs [19].

It would be too simple, however, to argue that 30% of newcomers fosters creativity. Both the increasing numbers of women in the historical profession and the rise of gender as a historical methodology relied on the broader political upheavals of the 1960s and ‘70. As numerous historians have told, women’s history and gender as an analytical category arose with the rekindling of feminism of the 1960s and ‘70s across Europe and the US, the US Civil Rights movement of the 1950s and ‘60s, and the general opening of history to new topics, such as social history [31, 32].

The origins of gender studies were also decidedly interdisciplinary. Gender as an analytical framework was fueled by historians, literary scholars, art critics, sociologists, anthropologists, primatologists, and more—all of whom built on each other’s insights and theoretical advances. Gender studies also drew heavily from activist work outside the academy, which included writings by Black, Indigenous, and queer activists, such as the Combahee River Collective, Gloria Anzaldúa, and Leslie Feinberg [33–35].

Women as a broad group are well-represented in the field of history. As the AHA documents, women overall are currently being hired slightly above their representation in the PhD pool. Since about 2005, the share of women earning PhDs has hovered around 45%, while women represent 49% of assistant professors hired for the 2017/18 academic year. Women represent 45% of associate professors, which also mirrors their representation among PhDs at the time of degree. At the level of full professor, however, women drop to 33% [36].

**Fig 8:**
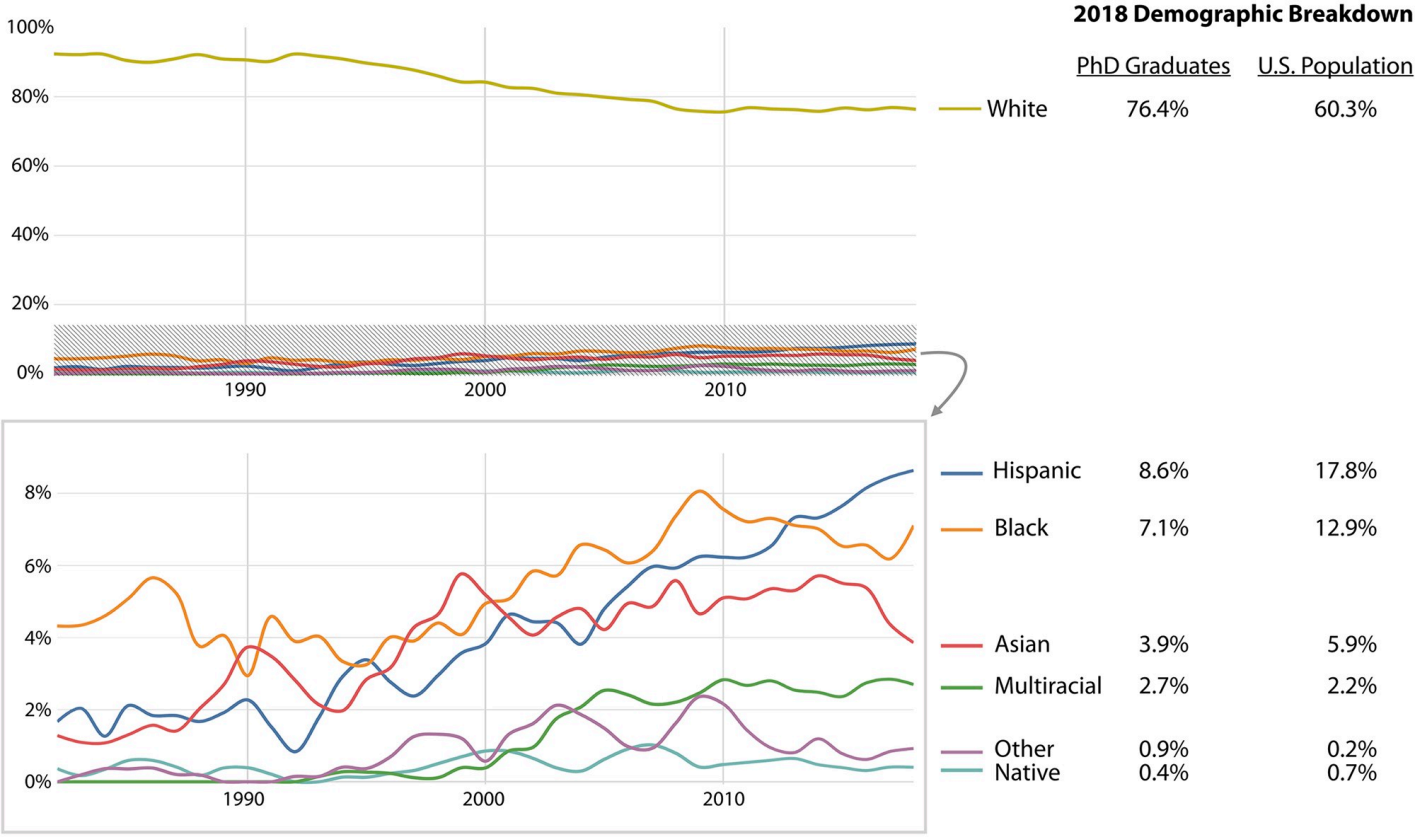
Demographics of women PhDs, by ethnicity. These categories are consistent with the categories used by the US Census and the NSF’s Survey of Earned Doctorates (SED) [19]). However, before the 1990s, “multiracial” was not available as a category on the SED, and multiracial people were listed under “other.” Today, only Native Hawaiian and other Pacific Islanders (NHOPI) fall in the “other” category. As such, for the US Census numbers listed above, we included the statistic for NHOPI with “other”.

But that is not the whole story. Data on the history profession from the National Science Foundation (NSF) demonstrate that white women have consistently earned 75–90% of all PhDs granted to women [19], while women of color are not represented at levels we might expect given their proportion of the US population [37]. Using categories provided by the US Census and the NSF, Black, Hispanic, Asian, and Native women are significantly underrepresented among history PhD graduates compared to the US population. For example, in 2018, Hispanic women represented 17.8% of all US women but only 8.6% of women History PhDs. Similarly, Black women represented 12.9% of all US women, but only 7.1% of women History PhDs [37]; see Fig 8. This source provides no data on women with disabilities or trans and genderqueer individuals. For similar statistics on men, see S8 Fig.

We are unable to trace women of color’s movement into the professorate because equivalent data on gender disaggregated by race (or race disaggregated by gender) are not available. Yet, from qualitative evidence, we know that women of color, many of whom pioneered intersectional historical approaches, have long been and continue to be marginalized in the historical discipline [38, 39].

While the historical profession still needs to make significant strides towards including marginalized scholars, gender analysis has, in many ways, been mainstreamed into the profession. The first women’s studies program was founded at San Diego State University in 1970 and followed quickly by dozens of others. The Berkshire Conference of Women’s Historians, founded already in 1930, became a national conference in 1973. The Association of Black Women Historians was founded six years later. These conferences fostered methodological advances in the field and were places where scholars strategized how best to institutionalize these approaches into History departments nationally [40].

A broader scholarly infrastructure emerged to support these changes. *Gender & History*, for example, was founded at the urging of Sue Corbett at Blackwell publishing in Oxford, England. Corbett, a highly successful editor, was promoted to head up the journals division in 1986 [41]. The same year that Corbett was promoted, Emory University established one of the first research centers on women’s studies (the Institute for Women’s Studies), appointed its first faculty in 1989, and accepted its first PhD students a year later [42]. Almost twenty years later, the American Academy of Arts and Sciences has reported that 283 departments across the United States have granted degrees in women’s and gender studies (WGS), with a total enrollment of 109,360 students in WGS courses in fall 2017 [43]. Women have also emerged as presidents of professional organizations. Nellie Neilson served as the first woman president of the AHA (an organization founded in 1884) in 1943. It was not until the 1980s, however, that women’s leadership took off. Anne Firor Scott won the Organization of American Historians presidency in 1984 and Natalie Zemon Davis the AHA presidency in 1987. Since 1987, the AHA has elected 13 women and 20 men presidents.

While this quantitative, longitudinal study suggests a positive correlation between the changing demographics and the types of questions asked in the field of history, the available tools did not allow us to employ intersectional approaches. How would our picture be changed by analyzing how gender and ethnicity interact, for example, or gender and sexual orientation, socioeconomic status, or geographic location? Nor can we answer the causal question of which came first, the openness of the discipline to new questions or the increase of women in these fields.

## Conclusions

We interpret our findings as indicating a symbiotic relationship in the discipline of history between “fixing the numbers” (increasing demographic representation in a field) and “fixing the knowledge” (opening a discipline to new research questions) [44, 45]. Numerous institutions seek to increase the numbers of underrepresented groups among their ranks. As this study has suggested, more is needed. Scholars distinguish three approaches to diversifying the academy: diversity in research teams, diversity in research methods, and diversity in research questions [46]. This study documents that to achieve equality in numbers also requires attention to what constitutes knowledge in a discipline.

## Supporting information

S1 File Contains supporting Text A through G and Tables A through I. (PDF)

S1 Data OSF repository for “diversifying history”. (PDF)

S1 Fig Four types of diagnostic values of correlated topic model. (TIF)

S2 Fig Decile plot for the topic “women and gender”. S2 Fig displays trends in women and men historians’ contributions to the “women and gender” topic over time, at various percentile ranges. The top row outlines developments for the bottom 10% articles with the lowest topic weight for the “women and gender” topic. The bottom row shows developments for the top 1% articles with the highest topic weight for the women and gender topic. The bar charts specify the relative representation of women (red) and men (blue) at each percentile range and show that women historians are vastly overrepresented in the top 1%, 5% and 10% articles with the highest topic weight for “women and gender.” Some 30% of all articles published by women score in the top decile for this topic.
(TIF)

S3 Fig Decile plot for the topic “body history”. S3–S5 and S7 Figs show the decile plots for the topics “body history,” “family and household,” “consumption and consumerism,” and “sexuality.” See the GitHub repository, “Percentile Plots,” last updated 19 May 2020. https://github.com/srisi/gender_history/blob/master/writeups/percentile_plots.md.
(TIF)

S4 Fig Decile plot for the topic “consumption and consumerism”. (TIF)

S5 Fig Decile plot for the topic “family and household”. (TIF)

S6 Fig Decile plot for the topic “sexuality”. (TIF)

S7 Fig Plots of overall topic weight and key terms for the “family and household” topic. S7 Fig reports historical trends in the overall topic weight (large panel) and key terms related to the “family and household” topic (smaller panels). The trend-line color in the large panel indicates the gender composition of authors focusing on the “family and household” topic at a given point in time—red signals that women are dominating the topic, blue signals that men are dominating the topic. The trend-line color in the smaller panels specify developments in the gender composition of the authors using key terms (family, children, women, marriage, household, parents) within the “family and household” topic at a given point in time. The trend-line color was determined based on the following formula: *avg_topic_weight(women) / (avg_topic_weight(women) + avg_topic_weight(men)*). The topic graph shows the average weight across all articles, while the term charts show the average term frequency across all charts. The numbers in Fig 7 include co-authored articles that were written by men only (50 articles, 4.3% of the dataset) or women only (53 articles, 0.5% of the dataset). However, it excludes co-authored articles written by a mix of men and women (302 articles, 2.9% of the dataset). Women also write fewer journal articles and books than they do dissertations (20).
(TIF)

S8 Fig Demographics of men PhDs, by ethnicity. (TIF)

## References

[1] Valantine HA, Collins FS. National Institutes of Health addresses the science of diversity. Proceedings of the National Academy of Sciences. 2015 Oct 6;112(40):12240–2. doi: 10.1073/pnas.1515612112 26392553 PMC4603507

[2] Nielsen MW, Alegria S, Börjeson L, Etzkowitz H, Falk-Krzesinski HJ, Joshi A, et al. Opinion: Gender diversity leads to better science. Proceedings of the National Academy of Sciences. 2017 Feb 21;114(8):1740–2. 10.1073/pnas.1700616114 PMC5338420 28228604

[3] Sulik J, Bahrami B, Deroy O. The diversity gap: when diversity matters for knowledge. Perspectives on Psychological Science. 2021 Oct:1–16. doi: 10.1177/17456916211006070 34606734

[4] Harding S. Whose science? Whose knowledge. Cornell University Press. 1991.

[5] Schiebinger L. Has feminism changed science? Cambridge, MA, Harvard University Press, 1999.

[6] Blei DM, Lafferty JD. Correlated topic models. In: Proceedings of the 18th International Conference on Neural Information Processing Systems. MIT Press, Cambridge, MA, USA, 2005. NIPS ‘05, pp. 147–154.

[7] Key EM, Sumner JL. You research like a girl: Gendered research agendas and their implications. PS: Political Science & Politics. 2019 Oct;52(4):663–8.

[8] Saraceno J. Disparities in a flagship political science journal? Analyzing publication -patterns in the Journal of Politics, 1939–2019. The Journal of Politics. 2020 Oct 1;82(4):e45–55.

[9] Light R. Gender inequality and the structure of occupational identity: The case of elite sociological publication. In: Mcdonald S, Ed. Research in the Sociology of Work. Emerald Group Publishing Limited, 2013; vol. 24, pp. 239–268.

[10] Dolado JJ, Felgueroso F, Almunia M. Are men and women-economists evenly distributed across research fields? Some new empirical evidence. SERIEs. 2012 Sep;3(3):367–93.

[11] Thelwall M, Bailey C, Tobin C, Bradshaw NA. Gender differences in research areas, methods and topics: Can people and thing orientations explain the results? Journal of informetrics. 2019 Feb 1;13(1):149–69.

[12] Nielsen MW, Börjeson L. Gender diversity in the management field: Does it matter for research outcomes?. Research Policy. 2019 Sep 1;48(7):1617–32.

[13] Hofstra B, Kulkarni VV, Galvez SM, He B, Jurafsky D, McFarland DA. The diversity–innovation paradox in science. Proceedings of the National Academy of Sciences. 2020 Apr 28;117(17):9284–91. 10.1073/pnas.1915378117 PMC7196824 32291335

[14] Davis HF. Beyond trans: Does gender matter? New York University Press; 2018 Sep 18.

[15] Mohammad SM. Gender gap in natural language processing research: Disparities in authorship and citations. arXiv preprint arXiv:2005.00962. 2020 May 3.

[16] Mihaljević H, Tullney M, Santamaría L, Steinfeldt C. Reflections on gender analyses of bibliographic corpora. Frontiers in Big Data. 2019 Aug 28;2:29. doi: 10.3389/fdata.2019.00029 33693352 PMC7931878

[17] Rasmussen KC, Maier E, Strauss BE, Durbin M, Riesbeck L, Wallach A, et al. The nonbinary fraction: looking towards the future of gender equity in astronomy. arXiv preprint arXiv:1907.04893. 2019 Jul 10.

[18] Matias JN. How to ethically and responsibly identify gender in large datasets. MediaShift. 2014. Available from: http://mediashift.org/2014/11/how-to-ethically-and-responsibly-identify-gender-in-large-datasets/

[19] National Center for Science and Engineering Statistics, National Science Foundation, Survey of Earned Doctorates (1958–2018); [cited 2021, May 23] (https://ncsesdata.nsf.gov/ids/sed).

[20] Christian D. The case for “Big History”. Journal of World History. 1991 Oct 1;2(2):223–38.

[21] Bonnel VE, Hunt L. Eds. Beyond the cultural turn: New directions in the study of society and culture. Berkeley: U of California P. 1999.

[22] Underwood T. Visualizing topic models. The Stone and the Shell. 2012. https://tedunderwood.com/2012/11/11/visualizing-topic-models/#technote.

[23] Smith BG. Gender I: From women’s history to gender history. In: Partner N, Foot S, Eds. The SAGE Handbook of Historical Theory. SAGE Publications, London, UK, 2013; pp. 266–281. http://sk.sagepub.com/reference/hdbk_historicaltheory

[24] Zinsser JP. Women’s history/feminist history. In: Partner N, Foot S, Eds. The SAGE Handbook of Historical Theory. SAGE Publications, London, UK, 2013; pp. 238–265. http://sk.sagepub.com/reference/hdbk_historicaltheory

[25] Townsend RB. The rise and decline of history specializations over the past 40 years. Perspectives on History. 2015. https://www.historians.org/publications-and-directories/perspectives-on-history/december-2015/the-rise-and-decline-of-history-specializations-over-the-past-40-years.

[26] Townsend RB. What’s in a label? Changing patterns of faculty specialization since 1975. Perspectives on History. 2007. https://www.historians.org/publications-and-directories/perspectives-on-history/january-2007/whats-in-a-label-changing-patterns-of-faculty-specialization-since-1975.

[27] Miller D. Material cultures: why some things matter. London: UCL Press, 1998.

[28] Munoz-Najar Galvez S, Heiberger R, McFarland D. Paradigm wars revisited: A cartography of graduate research in the field of education (1980–2010). American Educational Research Journal. 2020 Apr;57(2):612–52.

[29] Grey S, Tremblay M, Dahlerup D, Childs S, Krook ML. Do women represent women? Rethinking the "critical mass" debate. Politics & Gender. 2006. 2, 491–492.

[30] Townsend RB. The status of women and minorities in the history profession. Perspectives on history, American Historical Association. 2002. https://www.historians.org/publications-and-directories/perspectives-on-history/april-2002/the-status-of-women-and-minorities-in-the-history-profession.

[31] Scott JW. Gender and the politics of history. Columbia University Press; 1988 Mar 2.

[32] Cott NF. Faust DG. Recent directions in gender and women’s history. OAH Magazine of History, Volume 19, Issue 2, March 2005, Pages 4–5, doi: 10.1093/maghis/19.2.4

[33] Combahee River Collective. A black feminist statement. Women’s Studies Quarterly. 2014 Oct 1:271–80.

[34] Anzaldua G. Borderlands/La Frontera: the new Mestiza Aunt Lute Books. San Francisco, CA. 1987.

[35] Feinberg L. Transgender warriors: making history from Joan of Arc to Dennis Rodman. Beacon Press; 1997.

[36] Directory of History Departments and Organizations, American Historical Association; 2020 [cited 2020, April 8]. https://secure.historians.org/members/services/cgi-bin/memberdll.dll/openpage?wrp=search_institution.htm.

[37] US Census Bureau. 2018 Population estimates by age, sex, race and Hispanic origin. The United States Census Bureau; 2019 [cited 2021, May 23]. https://www.census.gov/newsroom/press-kits/2019/detailed-estimates.html.

[38] Lorde A. The uses of anger: Women responding to racism. 1981. https://www.blackpast.org/african-american-history/1981-audre-lorde-uses-anger-women-responding-racism/.

[39] Miller A. Townhouse notes: History has a race problem, and it’s existential. Perspectives on History. 2019. https://www.historians.org/publications-and-directories/perspectives-on-history/november-2019/townhouse-notes-history-has-a-race-problem-and-its-existential.

[40] Scott JW. The fantasy of feminist History. Duke University Press, 2011.

[41] Davidoff L. Katie Barclay interviews Leonore Davidoff, founding editor of Gender & History. 2012. https://onlinelibrary.wiley.com/pb-assets/assets/14680424/KB_-_Interview_with_LD_-_transcript.pdf.

[42] Women’s, Gender, and Sexuality Studies, Emory University, Our History. http://wgss.emory.edu/home/about-us.html.

[43] Porter AM, Pold J, White S. The state of women and gender studies in four-year colleges and universities (2017). American Academy of Arts and Sciences, 2020. https://www.amacad.org/sites/default/files/media/document/2020-05/hds3_women_and_gender_studies_profile.pdf.

[44] Schiebinger L, Schraudner M. Interdisciplinary approaches to achieving gendered innovations in science, medicine, and engineering1. Interdisciplinary Science Reviews. 2011 Jun 1;36(2):154–67.

[45] Nielsen MW, Bloch CW, Schiebinger L. Making gender diversity work for scientific discovery and innovation. Nature Human Behaviour. 2018 Oct;2(10):726–34. doi: 10.1038/s41562-018-0433-1 31406295

[46] Nielsen MW, Andersen JP, Schiebinger L, Schneider JW. One and a half million medical papers reveal a link between author gender and attention to gender and sex analysis. Nature human behaviour. 2017 Nov;1(11):791–6. doi: 10.1038/s41562-017-0235-x 31024130

