# Supplementary material for: Diversifying history: A large-scale analysis of changes in researcher demographics and scholarly agendas
Source: PLoS One. 2022 Jan 19;17(1):e0262027. doi: 10.1371/journal.pone.0262027 (PMC8769356; doi:10.1371/journal.pone.0262027)
Supplement: S1 File — (PDF) [file pone.0262027.s001.pdf]

S1 Text

Text A. Correlated topic models

We used Correlated Topic Modeling (CTM) to identify research areas and themes discussed in 10,367 full-text articles for the period 1951 to 2014 from a set of History journals published in the US. CTM is a natural language processing technique that uncovers latent dimensions in text corpora, i.e., "topics" as sets of terms weighted by their co-occurrence in the same documents. Prior work finds CTM to be superior to Latent Dirichlet Allocation in this context (particularly for held out log probability, which lets CTM cover a larger K value of topics better than LDA) [1].

The input to CTM is a document term matrix. In our case, the terms we use are n-grams (unigrams, bigrams, and trigrams). To generate this matrix we first stem words, remove stop words (overly common terms), numbers, punctuation, and words that are less than three characters and which occur in more than one document. This rendered our text into 356,140 unigrams, bigrams, or trigrams. (We retain low frequency terms because much of historical knowledge is contextually specific. We believe this retains the greatest nuance in the data, but also it comes with the cost of additional computing time and power.) CTM then identifies topics in this matrix by finding sets of terms weighted by their rate of co-occurrence. We identified the history topics most indicative and predictive of the terms used in our corpus through a variety of fit metrics (held-out likelihood, residuals, semantic coherence, and lower bound tests) from the Search K package in R (Appendix C). These metrics identify how different numbers of topics can result in sets of terms with greater/lesser internal consistency and predictive power. An optimal fit was found across these metrics for 90 topics.

CTM also produces an assessment of each document fed into the model, allowing us to interpret each document as a mixture of the topics generated. While CTM does not automatically label a topic, we name each topic based on frequently used words and exemplary journal articles that ranked highest in each topic. For example, the CTM generated a topic where the words "family," "children," "marriage," "household," and "parent" where frequently co-used. In addition, we looked at documents that heavily employed the topic to discern what the terms reflected. After manual inspection, we labeled this topic "family and households." Among the 90 generated topics, eight topics were associated with dataset noise, and therefore excluded from our analysis. The rest of topics were then categorized into significant history subfields and general approaches.

Text B. Fit metrics

Running a Correlated Topic Model requires that the researcher determines a value of K, or the number of topics. K is not automatically selected by the CTM, and instead, researchers need to find a number that optimizes the coherence and exclusivity of topics. If we set the number of topics too low, words describing topics might not sufficiently overlap (i.e., describing one topic); in addition, the scope of topic might be too broad to maintain the coherence of words. By contrast, if we extract too many topics, each topic might be coherent, but the same words can be adopted by many different topics. Thus, K value selection depends on how much coherence can be spared to maximize exclusivity, and vice versa.

To find the right number of topics that can balance coherence and exclusivity, we checked the internal validity of the model by increasing the number of topics from 60 to 120 topics in steps of 10 additional topics for each model. To check the internal validity, we used four

measures. Figure 1 above presents the results of these metrics for values of K ranging from 60 to 120. For these diagnostics, we used the function 'searchK' from 'stm' library in R.

First, held-out likelihood (top left in S1 Fig), sometimes called held-out log likelihood, is a measure to show the predictive power of topic models [2]. This method subsets 10% of the analyzed documents and holds out 50% of words in them. Then, it re-runs the same evaluative measures and compares them to the results without removing words. When held-out likelihood is high, it means the topic model has high predictive power. Second, residuals (top right) shows estimated residuals dispersion. Overdispersion means that we still need to add extra topics to absorb residuals [2]. Thus, we want to minimize our residual metric to increase our model's explanatory power. Third, semantic coherence (bottom left) is a metric that measures how much the words frequently used in the topic are co-occurring together [3]. For example, when "woman" and "movement" are the most probable words in the topic about feminism, the coherence of this topic is high when these two words are used simultaneously in the same document repeatedly. Thus, semantic coherence corresponds most closely with human interpretation of topic quality. As we evaluate the topic quality is good when the metric is high, we want to maximize it as much as possible without sacrificing the other metrics too much. Last, the lower bound (bottom right) represents the model's internal fit by measuring convergence. If the changes in convergence become less and less distinct as we raise the value of K, we can be satisfied that the model has converged. Here, we are not looking simply for maximization, but for the point where increasing K further provides diminishing returns.

Although we aim to find the flattened part in the curve where neither gains nor losses are found by adding more topics [2], our results in Figure 1 do not provide a clear signal. Instead, it shows that the constant advantage of using a higher K in held-out likelihood, residuals, and lower bound, and at the same time, the constant disadvantage higher K's have to semantic coherence. Therefore, we select a middle ground by choosing 90 topics that optimize semantic coherence without raising the other values too sharply. Additionally, the output for 90 topics covered the anticipated range of topics and subfields we expected to find in our dataset (e.g., topics historians expect to find).

Text C. Gender disambiguation

Inferring genders from names is a complex endeavor. Please see the body of the article for the limitations and ethical concerns associated with these methods. A name's association can reverse over time—"Leslie" was a "male" first name until the early twentieth century but has since become a "female" first name in the United States [4]. Additionally, the association between names and genders depends on the country. Take "Jan," for example. Per Social Security Administration (SSA) data, "Jan" is a "female" name in 71% of cases in the United States [5]. In the Netherlands and Germany, however, "Jan" is almost always a "male" name. Our goal in inferring genders from first names was to automate the process as much as possible, while taking care to identify ambiguous cases and labeling those by hand. As we mention in the body of the article, automated gender inference is used not to predict or determine an individual's gender, but to analyze gender dynamics in the historical profession as a whole.

To achieve this goal, we used two complementary methods: comparison with unique names in the dataset provided by the SSA and with the Python "gender-guesser" library [6]. With both of these methods, if the name was consistently "male" or "female," we took that result. If, however, the name was ambiguous because the first name presented only as an initial ("H. Michael") or because the name was unisex ("Jaime Elizabeth"), we also examined the middle

name. If the middle name was classified as "male" or "female," we returned that as the result. If neither name could be classified, we returned "ambiguous" and labeled the name manually.

For our first method—inferring gender using SSA data—we grouped results into five categories. If a name was given to women in 95% or more of all cases, we labeled it "female"—and vice versa for men. If a name was given to women or men between 85% and 95%, we labeled it "mostly female" or "mostly." We tagged all other names "ambiguous."

In addition to SSA data, we used the Python "gender-guesser" library. This library is an off the shelf tool to infer gender from names. While the provenance of the data that it uses is hard to track, the underlying dataset was compiled by Jörg Michael, a German computer scientist, in 2008. A recent study comparing five similar libraries and services found that the "gender-guesser" had the lowest misclassification rate, meaning that it, although it labels many names "ambiguous," it rarely assigned the wrong gender to a name [7; 8]. An additional advantage of the gender-guesser library, for our purposes, is that it includes data not just from the United States but also from most European as well as some Asian countries. With SSA data, for example, both "Jean" and "Patrice" are labeled "female," but the gender-guesser library tags both of them "ambiguous" because they can also be "male" names in France. Hence, the gender-guesser library helped us question some of the results obtained with SSA data and identify names that should be reexamined.

Once we had the inferred genders using both SSA data and the gender-guesser library, we compared them. If one method presumed a person to be "male" and the other classified them as "male" or "mostly male," we classified that person "male"—and vice versa for women. In any other case, for example where the two methods disagreed or when one method labeled the gender "ambiguous," we tried to identify the author's gender manually via an internet search, using, for example, faculty web pages or news releases. If we still could not identify the gender of an author, we labeled the gender "unknown."

Overall, for the journal dataset, we were able to identify 6821 authors (84.3%) automatically, using SSA data and the "gender-guesser," and 1044 authors (12.8%) by tagging them manually, leaving 231 authors (2.9%) with unknown genders. For the dissertation dataset, we identified the genders of 20771 authors (97.4%) automatically and 319 authors (1.5%) by tagging them manually, leaving 230 authors (1.1%) with unknown genders. Our automatic taggers were more successful on the dissertation dataset for a number of reasons. First, many journal articles include an author's initials instead of full name. Second, the dissertation dataset more often includes middle names than the journal dataset. Finally, the journal dataset is more international than the dissertation dataset, rendering the US SSA dataset less useful.

Text D. Gini coefficients

To calculate the gini coefficient for a topic and year, we first formed a document-topic matrix (A) for that year. Next, we calculated a co-occurrence matrix C by multiplying A with its own transpose (C = A * A.T). Finally, we calculated the gini coefficients for each topic by using the rows in C representing each topic.

Text E. Overrepresented topics among women

To calculate the topics that are overrepresented among women writing about military history, we first selected only articles scoring in the top 10% for the military history. Then we formed two sub-corpora, one for men (830 articles) and one for women (134 articles) and compared them using Dunning's log-likelihood statistic [9]. In the women's corpus, the five most over-

represented topics were "Women and Gender," "Family," "Film, Memory, and Documentation," "Body History," and "Cultural History of the Civil War."

Text F. Gender and women's history in the cultural history of war

Specifically, gender and women's history is well represented in such titles as "Walking the Streets in a Way No Decent Woman Should"; "Women Police in World War I"; "French Volunteer Nursing and the Myth of War Experience in World War I"; "Sex, Citizenship, and the Nation in World War II Britain"; "Altars of Sacrifice: Confederate Women and the Narratives of War"; Black Professionals and Race Consciousness: Origins of the Civil Rights Movement"; and "Battle Time: Gender, Modernity, and Confederate Hospitals." For additional explanation, see Risi, "Differences within Topics" (last modified 25 May 2020), https://github.com/srisi/gender_history/blob/master/writeups/differences_within_topics.md

Text G. Analysis of how dissertations load on the 90 journal topics

To best capture how dissertations relate to the journal topics, we first aligned the vocabulary of the dissertation dataset to the journal topic dataset vocabulary. This means that terms present in the dissertation dataset, but not the journal dataset, were discarded. Among 10,000 high frequency terms in dissertation abstracts (ProQuest Dissertation & Theses database), 94% of terms were found in journal articles (JSTOR database).

Given that the two corpora—dissertations and journal articles—are similar in the usage of terms, we apply our Correlated Topic Model (CTM) results generated from the journal articles to dissertation abstracts. In our CTM results from journal articles, each term has a load (weighted score) to the 90 topics. Using the CTM results, we apply the information of loads from terms to topics to the corpus of dissertations. For example, if "woman" has high weight to the gender topic from our CTM results of journal articles, we can infer that a doctoral dissertation using "woman" multiple times will have high load on the gender topic. In this manner, we can use the same topics to analyze the dissertations as the journals, while still allowing an overview of how similar or dissimilar the datasets are.

Table A. Top 100 articles with the highest weight for the topic "women and gender"

Topic Weight	Year	Author Gender	Title
0.46	1994	female	"To Educate Women into Rebellion": Elizabeth Cady Stanton and the Creation of a Transatlantic Network of Radical Suffragists
0.446	1993	female	Beyond the Feminine Mystique: A Reassessment of Postwar Mass Culture, 1946- 1958
0.431	1990	female	Liberal Ideology, Sexual Difference, and the Lives of Women: Recent Works in British History
0.431	1987	female	Working Women, Class Relations, and Suffrage Militance: Harriot Stanton Blatch and the New York Woman Suffrage Movement, 1894-1909
0.421	1981	female	Reflections on Twentieth-Century American Women's History

0.421	1989	female	What's in a Name? The Limits of 'Social Feminism;' or, Expanding the Vocabulary of Women's History
0.419	1988	female	Separate Spheres, Female Worlds, Woman's Place: The Rhetoric of Women's History
0.399	2007	female	Rethinking the Socialist Construction and International Career of the Concept "Bourgeois Feminism"
0.391	1982	female	Expanding the Past: Recent Scholarship on Women in Politics and Work
0.38	1993	unknown	The Changing Critical Fortunes of the Second Sex
0.379	1986	female	The Doctrine of "Equality in Difference" for Women: A Case Study of Male Feminism in Nineteenth-Century French Thought
0.371	1992	female	The Manly Pursuit of a Partnership between the Sexes: The Debate over YMCA Programs for Women and Girls, 1914-1933
0.369	1987	female	Outgrowing the Compact of the Fathers: Equal Rights, Woman Suffrage, and the United States Constitution, 1820-1878
0.369	1987	unknown	Women of Their Time: The Growing Recognition of the Second Sex in Victorian and Edwardian England
0.365	1987	female	Century of Struggle, Decades of Revision: A Retrospective on Eleanor Flexner's Suffrage History
0.356	1986	female	Gender: A Useful Category of Historical Analysis
0.351	1989	female	Women's Labor History, 1790-1945
0.348	1984	female	The Domestication of Politics: Women and American Political Society, 1780-1920
0.345	1992	unknown	The "Other" as Political Symbol: Images of Indians in the Woman Suffrage Movement
0.342	1988	female	The Professionalization of Benevolence: Evangelicals and Social Workers in the Florence Crittenton Homes, 1915 to 1945
0.342	1974	female	The New Woman: Changing Views of Women in the 1920s
0.333	2012	female	The Big Tent of U.S. Women's and Gender History: A State of the Field
0.331	1990	female	Gender
0.331	1976	male	Cookbooks and Law Books: The Hidden History of Career Women in Twentieth Century America
0.322	2012	female	Can We Have a Total American History? A Comment on the Achievements of Women's and Gender History

0.321	1989	female	Working-Class Feminism and the Family Wage Ideal: The Seattle Debate on Married Women's Right to Work, 1914-1920
0.32	1969	female	New Approaches to the Study of Women in American History
0.317	1990	mixed	Womanly Duties: Maternalist Politics and the Origins of Welfare States in France, Germany, Great Britain, and the United States, 1880-1920
0.316	1984	unknown	The Contribution of Women to Modern Historiography in Great Britain, France, and the United States, 1750-1940
0.312	2012	female	The Impact of Racial and Sexual Politics on Women's History
0.307	1971	male	Comment: ["Women in the Russian Radical Movement" and "Women Reformers and American Culture, 1870-1930"]
0.306	1978	female	Woman's Place: A Critical Review of Anthropological Theory
0.302	2012	female	Gender, Generations, Leadership
0.296	1992	female	"Nous Autres": Reading, Passion, and the Creation of M. Carey Thomas
0.296	1990	female	Finding a Cure for War: Women's Politics and the Peace Movement in the 1920s
0.294	1999	female	Working on White Womanhood: White Working Women in the San Francisco Anti-Chinese Movement, 1877-1890
0.294	1988	female	"We Are Engaged as a Band of Sisters": Class and Domesticity in the Washingtonian Temperance Movement, 1840-1850
0.294	2001	male	"A New World for Women"? Abortion Law Reform in Britain during the 1930s
0.293	2000	female	"The Best or None!" Spinsterhood in Nineteenth-Century New England
0.291	1986	female	"Moral Suasion Is Moral Balderdash": Women, Politics, and Social Activism in the 1850s
0.285	1994	female	"Walking the Streets in a Way No Decent Woman Should": Women Police in World War I
0.284	1992	female	Introduction: History and Feminist Theory, or Talking Back to the Beadle
0.283	1962	male	The Split of Feminist Movement in 1869
0.283	2009	female	Sons, Daughters, and Patriarchy: Gender and the 1968 Generation
0.283	1990	male	Political Style and Women's Power, 1830-1930

0.283	1987	unknown	Class and Patriarchy as Competing Paradigms for the Study of Middle Eastern Women
0.279	1982	female	Women in the Professions: A Research Agenda for American Historians
0.279	1995	female	Beyond Complicity versus Resistance: Recent Work on Gender and European Imperialism
0.275	2002	female	From Mexico to Copenhagen to Nairobi: The United Nations Decade for Women, 1975-1985
0.274	2008	female	A History of "Gender"
0.27	1971	female	Women Reformers and American Culture, 1870-1930
0.27	1982	female	Zenanas and Girlless Villages: The Ethnology of American Evangelical Women, 1870-1910
0.268	1984	female	The Charitable and the Poor: The Emergence of Domestic Politics in Augusta, Georgia, 1860-1880
0.267	2012	male	"Russian Blonde in Space": Soviet Women in the American Imagination, 1950-1965
0.267	1991	female	Black and White Visions of Welfare: Women's Welfare Activism, 1890-1945
0.265	1999	female	Feminism, Social Science, and the Meanings of Modernity: The Debate on the Origin of the Family in Europe and the United States, 1860-1914
0.264	2012	female	Gender Identity and the Gendered Process
0.264	2010	female	The Incorporation of American Feminism: Suffragists and the Postbellum Lyceum
0.262	2002	female	Rape without Women: Print Culture and the Politicization of Rape, 1765-1815
0.262	1975	female	Leadership and Tactics in the American Woman Suffrage Movement: A New Perspective from Massachusetts
0.261	2001	female	Shrinking Violets and Caspar Milquetoasts: Shyness and Heterosexuality from the Roles of the Fifties to "The Rules" of the Nineties
0.26	2008	female	The Three Ages of Joan Scott
0.255	2005	female	Incarnations and Practices of Feminine Rectitude: Nineteenth-Century Gymnastics for U.S. Women
0.254	2008	female	Unanswered Questions
0.254	2010	female	Writing Women's Lives: One Historian's Perspective
0.253	1983	female	Labor's True Woman: Domesticity and Equal Rights in the Knights of Labor
0.251	1981	mixed	The Limits of Suffragist Behavior: Legalism and Militancy in France, 1876-1922

0.245	2001	male	Gender and Working Class Identity in Britain during the 1950s
0.243	2004	male	"The Forgetfulness of Sex": Devotion and Desire in the Courtship Letters of Angelina Grimke and Theodore Dwight Weld
0.243	1988	female	Women and Capitalism: Oppression or Emancipation? A Review Article
0.243	2004	female	Policing Male Heterosexuality: The Reformation of Manners Societies' Campaign against the Brothels in Westminster, 1690-1720
0.242	1986	female	Between Two Worlds: Business Women in a Chicago Boarding House 1900-1930
0.242	1982	female	Saint-Simonian Men/Saint-Simonian Women: The Transformation of Feminist Thought in 1830s' France
0.241	1980	male	The History of European Women: A Critical Survey of Recent Research
0.241	1984	female	Women in Groups: An Analysis of Women's Benevolent Organizations in New York and Boston, 1797-1840
0.24	1984	female	Depopulation, Nationalism, and Feminism in Fin-de-Siècle France
0.239	1994	female	Constructing Internationalism: The Case of Transnational Women's Organizations, 1888-1945
0.239	1982	female	Sexuality in Nineteenth-Century America: Behavior, Ideology, and Politics
0.237	1991	female	"The Anchor of My Life": Middle-Class American Mothers and College-Educated Daughters, 1880-1920
0.234	1991	mixed	The Politics of Gender and the Making of the Welfare State, 1900-1940: A Comparative Perspective
0.233	2007	female	World History and the History of Women, Gender, and Sexuality
0.231	1985	mixed	Female Ballots: The Impact of the Nineteenth Amendment
0.231	2004	female	Global Feminism and Postwar Reconstruction: The World YWCA Visitation to Occupied Japan, 1947
0.23	1990	female	A Call for Comparisons
0.23	1995	female	The Woman's Room: Some Aspects of Gender Relations in Tenochtitlan in the Late Pre-Hispanic Period
0.229	1996	female	French Volunteer Nursing and the Myth of War Experience in World War I
0.226	1986	female	Women's Rights and Society's Needs: Japan's 1931 Suffrage Bill

0.226	2010	female	"Those by Whose Side We Have Labored": American Jewish Women and the Peace Movement between the Wars
0.225	1999	male	Prostitutes in History: From Parables of Pornography to Metaphors of Modernity
0.225	1989	female	Gender Systems in Conflict: The Marriages of Mission-Educated Chinese American Women, 1874-1939
0.225	1982	female	The Pursuit of Married Love: Women's Attitudes toward Sexuality and Marriage in Great Britain, 1918-1939
0.223	1996	female	Embracing the Status Quo: French Communists, Young Women and the Popular Front
0.221	1972	male	Coeducation of the Sexes at Oberlin College: A Study of Social Ideas in Mid-Nineteenth-Century America
0.221	1980	female	A "New Frontier" for Women: The Public Policy of the Kennedy Administration
0.22	1996	female	Comment: Native American Women's Responses to Christianity
0.22	2008	unknown	Chinese History: A Useful Category of Gender Analysis
0.22	2002	female	Walking on the Periphery: Gender and the Discourse of Modernization
0.22	2002	female	Rescuing Women and Children
0.219	2000	mixed	Teaching Gender History to Secondary School Students
0.216	1969	male	Anna Howard Shaw: New Approaches to Feminism

Table B. Ten most distinctive terms by frequency score for women-authored articles that mention the term "gender" at least ten times (174 documents)

Term	Dunning Log-Likelihood Statistic	Frequency Score	Term Frequency, Women Historians	Term Frequency, Men Historians
feminist	620.7	0.15	0.470%	0.086%
feminism	385.3	0.18	0.336%	0.074%
Chinese	414.3	0.92	0.385%	0.092%
welfare	111.8	0.31	0.241%	0.109%
movement	175.0	0.33	0.453%	0.223%
women	2556.4	0.34	7.429%	3.843%
mothers	90.1	0.35	0.284%	0.150%
rights	92.1	0.37	0.410%	0.246%
woman	207.2	0.38	1.041%	0.646%
home	99.1	0.38	0.512%	0.320%

Table B reports the distinctive terms addressed by women historians in articles that mention "gender" at least ten times.

Table C. Ten most distinctive terms by frequency score for men-authored articles that mention the term "gender" at least ten times (93 documents)

Term	Dunning Log-Likelihood Statistic	Frequency Score	Term Frequency, Women Historians	Term Frequency, Men Historians
gay	112.4	0.73	0.056%	0.154%
masculinity	195.8	0.71	0.140%	0.337%
Americans	118.6	0.67	0.142%	0.288%
him	193.0	0.67	0.232%	0.471%
service	125.8	0.67	0.155%	0.312%
black	352.2	0.67	0.439%	0.882%
he	507.6	0.64	0.104%	0.182%
united	136.1	0.63	0.322%	0.546%
his	502.4	0.62	0.135%	0.223%
war	220	0.62	0.616%	1.001%

Table C reports the distinctive terms addressed by men historians in articles that mention "gender" at least ten times.

Table D. Ten most distinctive terms for articles in women's and gender history published from 1970-1989

Term	Dunning Log-Likelihood Statistic	Frequency Score	Term Frequency, 1970-1989	Term Frequency, 1990-2009
women	2419.0	0.59	4.416%	3.079%
work	887.5	0.62	0.989%	0.617%
family	862.0	0.62	0.896%	0.549%
percent	791.4	0.69	0.352%	0.158%
medical	606.7	0.70	0.244%	0.104%
age	512.3	0.65	0.346%	0.185%
table	508.5	0.73	0.159%	0.059%
married	455.4	0.66	0.287%	0.150%
data	403.6	0.72	0.142%	0.056%
household	383.6	0.66	0.228%	0.116%

Table D juxtaposes the most distinctive terms used by the top 10% articles with the greatest topic weight for "women and gender" in the periods 1970 to 1989. This is a comparison of the most distinctive terms by Dunning Log-Likelihood statistic used by the 10% articles with the greatest topic weight for "women and gender" in the periods 1970 to 1989 (275 documents) and 1990 to 2009 (650 documents).

Table E. Ten most distinctive terms for articles in women's and gender history published from 1990-2009

Term	Dunning Log-Likelihood Statistic	Frequency Score	Term Frequency, 1970-1989	Term Frequency, 1990-2009
gender	1534.9	0.59	0.172%	0.501%
white	1046.4	0.62	0.162%	0.413%
African	864.9	0.62	0.060%	0.218%
race	863.1	0.69	0.047%	0.193%
black	782.4	0.70	0.156%	0.363%
war	714.8	0.65	0.237%	0.466%
racial	688.8	0.73	0.025%	0.130%
nation	557.4	0.66	0.050%	0.161%
British	470.5	0.72	0.062%	0.169%
colonial	451.8	0.66	0.084%	0.200%

Table E juxtaposes the most distinctive terms used by the top 10% articles with the greatest topic weight for "women and gender" in the periods 1990 to 2009.

Table F. The top 5 overrepresented topics for students with a man advisor, dissertations (1990 to 2015, 9633 documents)

Topic	Dunning Log-Likelihood Statistic	Frequency Score	Average Topic Weight, Students with Female Advisors	Average Topic Weight, Students with Male Advisors
Military History	4926.9	0.36	1.739%	3.057%
Political History of the Cold War	3896.1	0.28	0.427%	1.087%
Political History (Revolutions)	1738.3	0.45	6.232%	7.547%
German and Austro-Hungarian Diplomatic History	1553.4	0.27	0.146%	0.395%
Islamic History	1214.7	0.37	0.473%	0.812%

We identified overrepresented topics using Dunning's log-likelihood statistics, comparing students with women advisors to those with male advisors. Tables F and G report the top 5 overrepresented topics for students with a man advisor and woman advisor, respectively, for dissertations submitted from 1990 to 2015.

Table G. The top 5 overrepresented topics for students with a woman advisor, dissertations (1990 to 2015, 3180 documents)

Topic	Dunning Log-Likelihood Statistic	Frequency Score	Average Topic Weight, Students with Female Advisors	Average Topic Weight, Students with Male Advisors
Women and Gender	27239.2	0.74	5.257%	1.803%
Consumption and Consumerism	5388.9	0.69	1.721%	0.785%
Cultural Turn	4063.4	0.59	5.216%	3.644%
Body History	3279.8	0.67	1.285%	0.641%
Family	2678.5	0.63	1.629%	0.948%

Table H. Top 10 dissertation topics that are overrepresented among dissertation writers (graduation dates between 1980 and 1999) *with* students (1306 documents)

Topic	Dunning Log-Likelihood Statistic	Frequency Score	Average Topic Weight, Dissertation Writers *with* Descendants	Average Topic Weight, Dissertation Writers *without* Descendants
Cultural Turn	1808.7	0.61	3.043%	1.968%
20th Century Labor History	1617.7	0.61	2.785%	1.811%
19th Century African American History	780.8	0.59	1.848%	1.286%
Rural Social History	764.6	0.56	3.590%	2.789%
Women and Gender	747.3	0.57	2.899%	2.192%
Political History (Revolutions)	567.4	0.53	8.398%	7.306%
Slavery in the Americas	474.6	0.60	0.913%	0.609%
Sociology and History	451.0	0.53	8.168%	7.204%
Colonies and Empires	396.2	0.56	2.022%	1.587%
East Asian History	384.8	0.58	1.017%	0.722%

To examine associations between student's dissertation topics and their likelihood of later having students of their own, we first formed a corpus of all dissertation abstracts of historians who finished their PhDs in the 1980s and '90s. Next, we used Dunning's log-likelihood statistic to compare dissertation abstracts of researchers with students (Table H) to those without students (Table I).

Table I. Top 5 topics that are overrepresented among dissertation writers (graduation dates between 1980 and 1999) *without* students (11454 documents)

Topic	Dunning Log-Likelihood Statistic	Frequency Score	Average Topic Weight, Dissertation Writers *with* Descendants	Average Topic Weight, Dissertation Writers *without* Descendants
Organizations	1794.5	0.38	1.730%	2.813%

Education	1428	0.39	1.649%	2.579%
Christianity	1263.6	0.42	2.675%	3.748%
Political History of the Cold War	1249.8	0.35	0.688%	1.286%
Military History	1238.3	0.40	1.754%	2.634%
Historiography (Archives)	670.9	0.44	2.970%	3.767%
20th Century British Foreign Policy	649.0	0.34	0.327%	0.627%
British Early Modern Political History	520.7	0.37	0.429%	0.723%
19th Century U.S. Political History	437.1	0.37	0.336%	0.576%
U.S. Political History	424.3	0.43	1.464%	1.913%

References

1. Blei DM, Lafferty JD. A correlated topic model of science. The Annals of Applied Statistics. 2007; 1, no. 1, pp. 17-35.

2. Roberts ME, Stewart BM, Tingley D. stm: R package for structural topic models. Journal of Statistical Software. 2014; 10, no. 2: 1-40.

3. Mimno D, Wallach HM, Talley E, Leenders M, McCallum A. Optimizing semantic coherence in topic models. Proceedings of the Conference on Empirical Methods in Natural Language Processing, Association for Computational Linguistics. 2011, July 27: 262-272.

4. Blevins C, Mullen L. Jane, John … Leslie? A historical method for algorithmic gender prediction. Digital Humanities Quarterly. 2015; 9, no 3.

5. Data from Social Security Administration. Baby names from social security card applications – National data. [cited 2020, June 14]. Available from: https://catalog.data.gov/dataset/baby-names-from-social-security-card-applications-national-level-data

6. Python gender-guesser library. [cited 2020, June 14]. Available from: https://github.com/lead-ratings/gender-guesser.

7. For the dataset, see Michael J. Dictionary of first names and gender. [cited 2020, June 14]. Available from: ftp://ftp.heise.de/pub/ct/listings/0717-182.zip

8. For the comparison of name-to-gender inference services, see Santamaría L, Mihaljević H. Comparison and benchmark of name-to-gender inference services. PeerJ Computer Science. 2018; 4, e. 156. [cited 2020, June 14]. Available from: https://peerj.com/articles/cs-156.pdf

9. Dunning T. Accurate methods for the statistics of surprise and coincidence. Computational Linguistics. 1993, March; 19, no. 1: 61–74.
