# Supplementary material for: Diversifying history: A large-scale analysis of changes in researcher demographics and scholarly agendas
Source: PLoS One. 2022 Jan 19;17(1):e0262027. doi: 10.1371/journal.pone.0262027 (PMC8769356; doi:10.1371/journal.pone.0262027)
Supplement: S1 Data — (PDF) [file pone.0262027.s002.pdf]

S1 Data

"Differences within Topics: Gender Differences in Articles Mentioning Gender at least 10 Times" within OSF Repository for "Diversifying History" (DOI: 10.17605/OSF.IO/V4YSH).

This file (last modified 25 May 2020) details differences in how men and women write about topics.

https://github.com/srisi/gender_history/blob/master/writeups/differences_within_topics.md

All data, tables, and supplemental write-ups not mentioned in the above figures are included in this OSF repository (DOI: 10.17605/OSF.IO/V4YSH). This includes iPython notebooks and R scripts.
