# Supplementary figures and images for: Diversifying history: A large-scale analysis of changes in researcher demographics and scholarly agendas
Source: PLoS One. 2022 Jan 19;17(1):e0262027. doi: 10.1371/journal.pone.0262027 (PMC8769356; doi:10.1371/journal.pone.0262027)

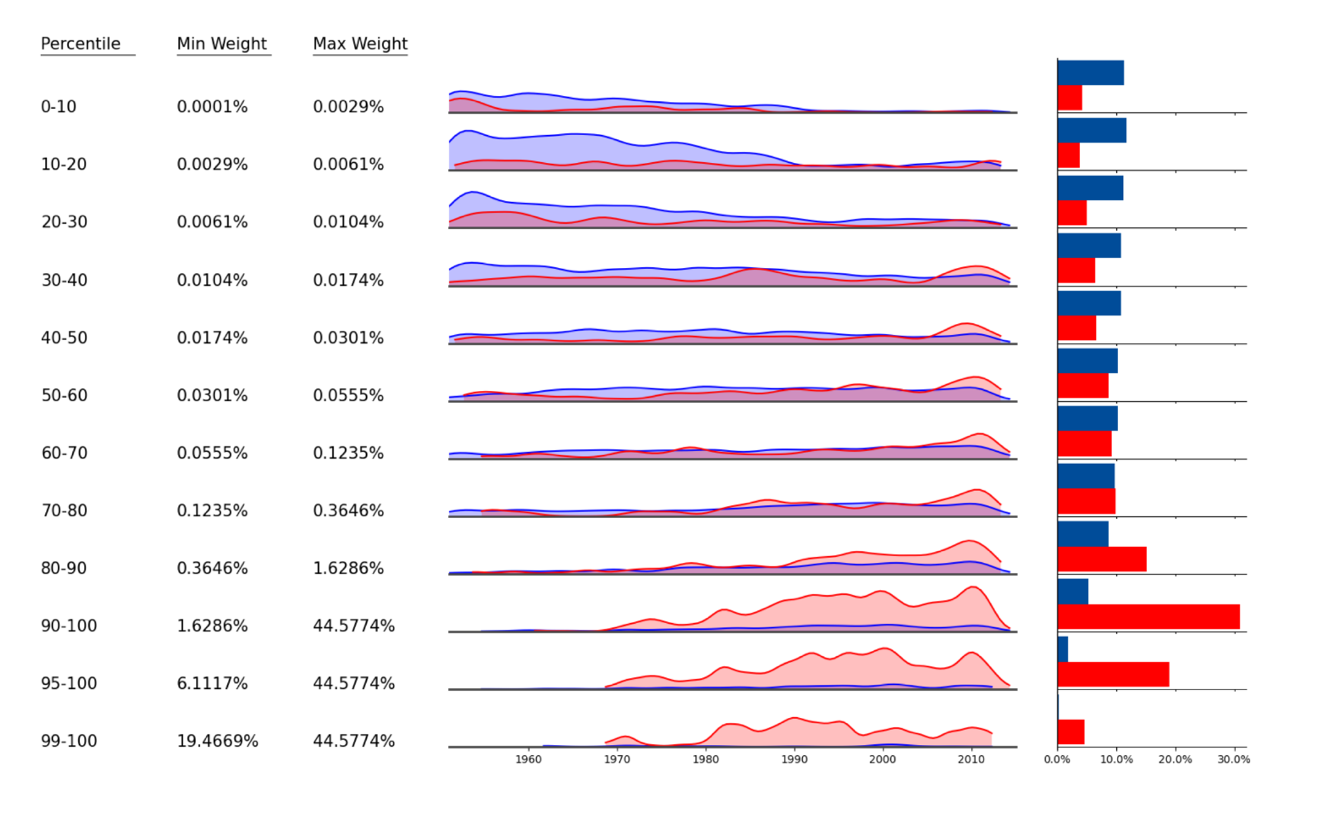

Supplement: S2 Fig — displays trends in women and men historians’ contributions to the “women and gender” topic over time, at various percentile ranges. The top row outlines developments for the bottom 10% articles with the lowest topic weight for the “women and gender” topic. The bottom row shows developments for the top 1% articles with the highest topic weight for the women and gender topic. The bar charts specify the relative representation of women (red) and men (blue) at each percentile range and show that women historians are vastly overrepresented in the top 1%, 5% and 10% articles with the highest topic weight for “women and gender.” Some 30% of all articles published by women score in the top decile for this topic. (TIF) [file pone.0262027.s004.tif]

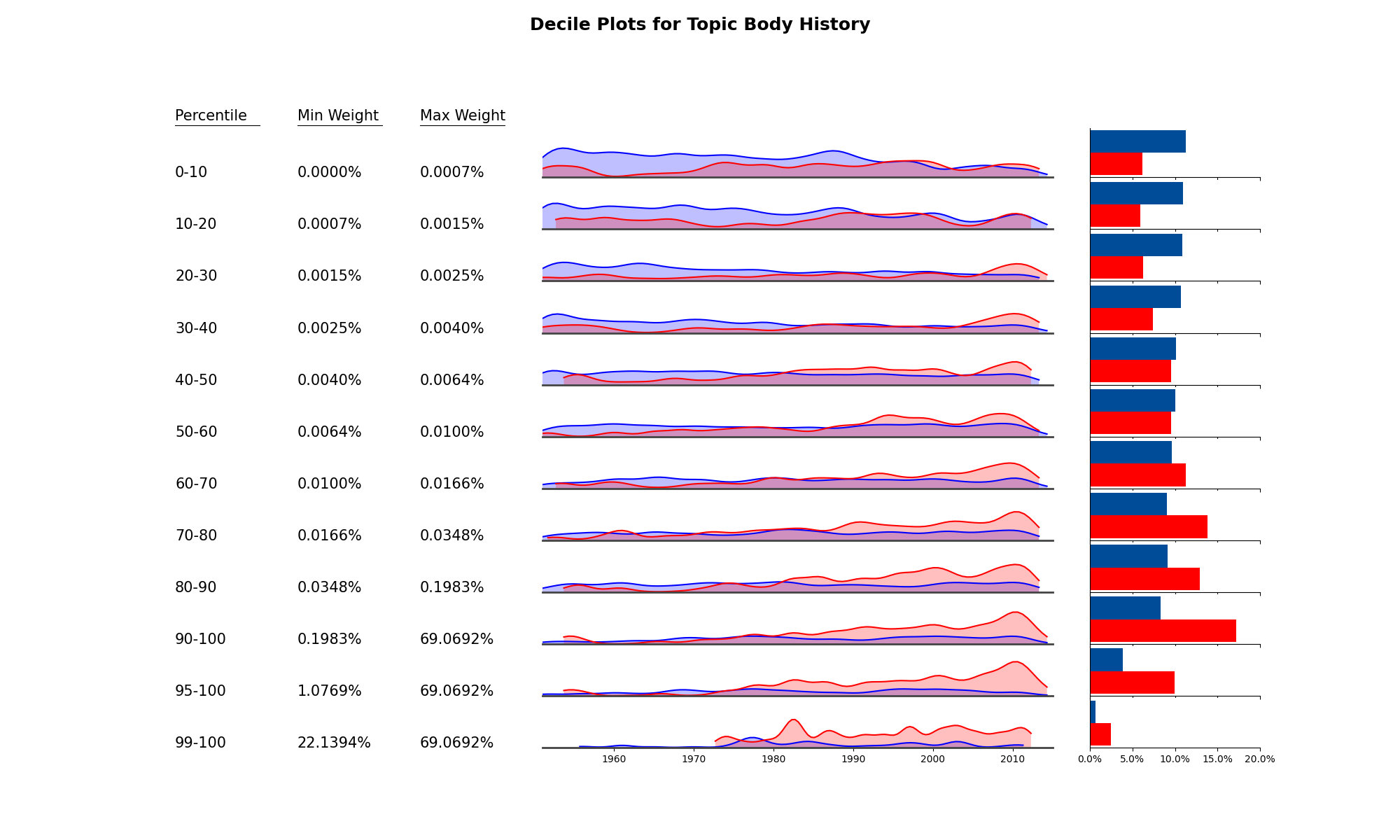

Supplement: S3 Fig — S3–S5 and S7 Figs show the decile plots for the topics “body history,” “family and household,” “consumption and consumerism,” and “sexuality.” See the GitHub repository, “Percentile Plots,” last updated 19 May 2020. https://github.com/srisi/gender_history/blob/master/writeups/percentile_plots.md. (TIF) [file pone.0262027.s005.tif]

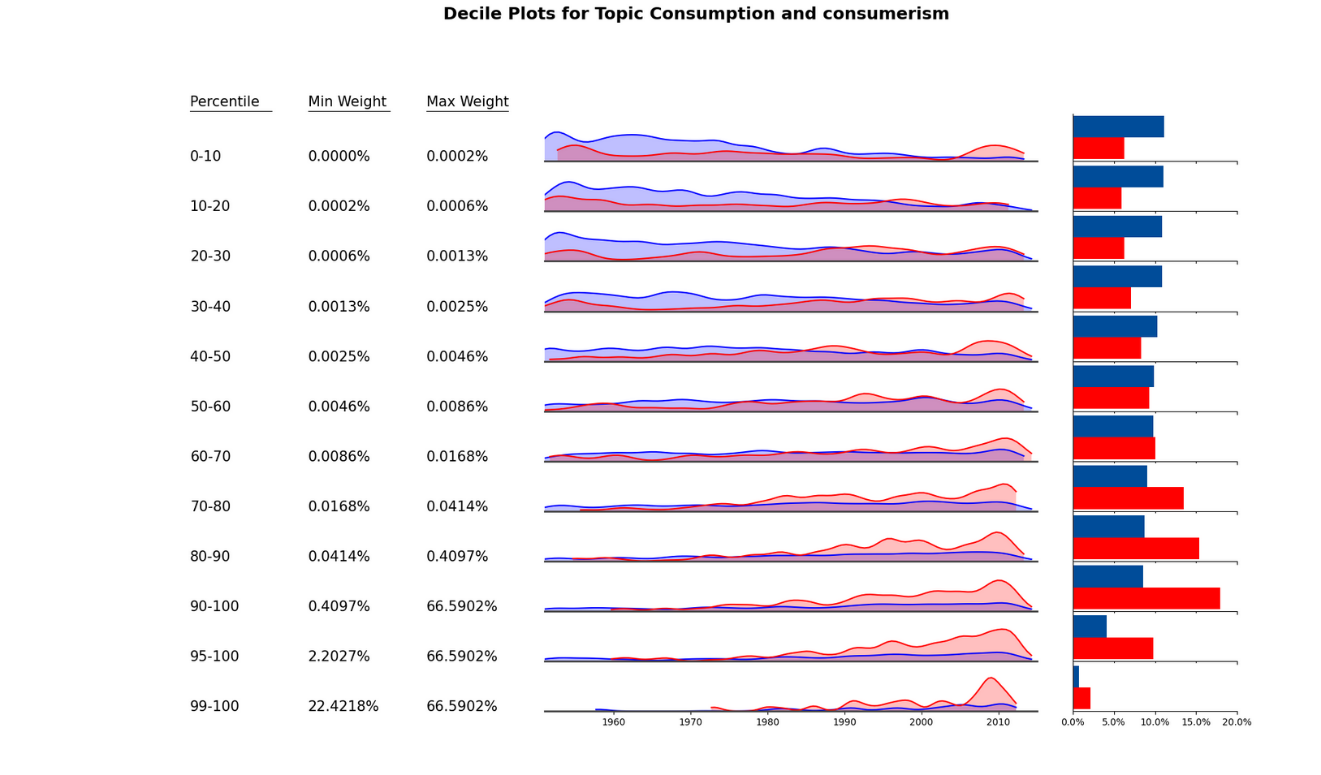

Supplement: S4 Fig — (TIF) [file pone.0262027.s006.tif]

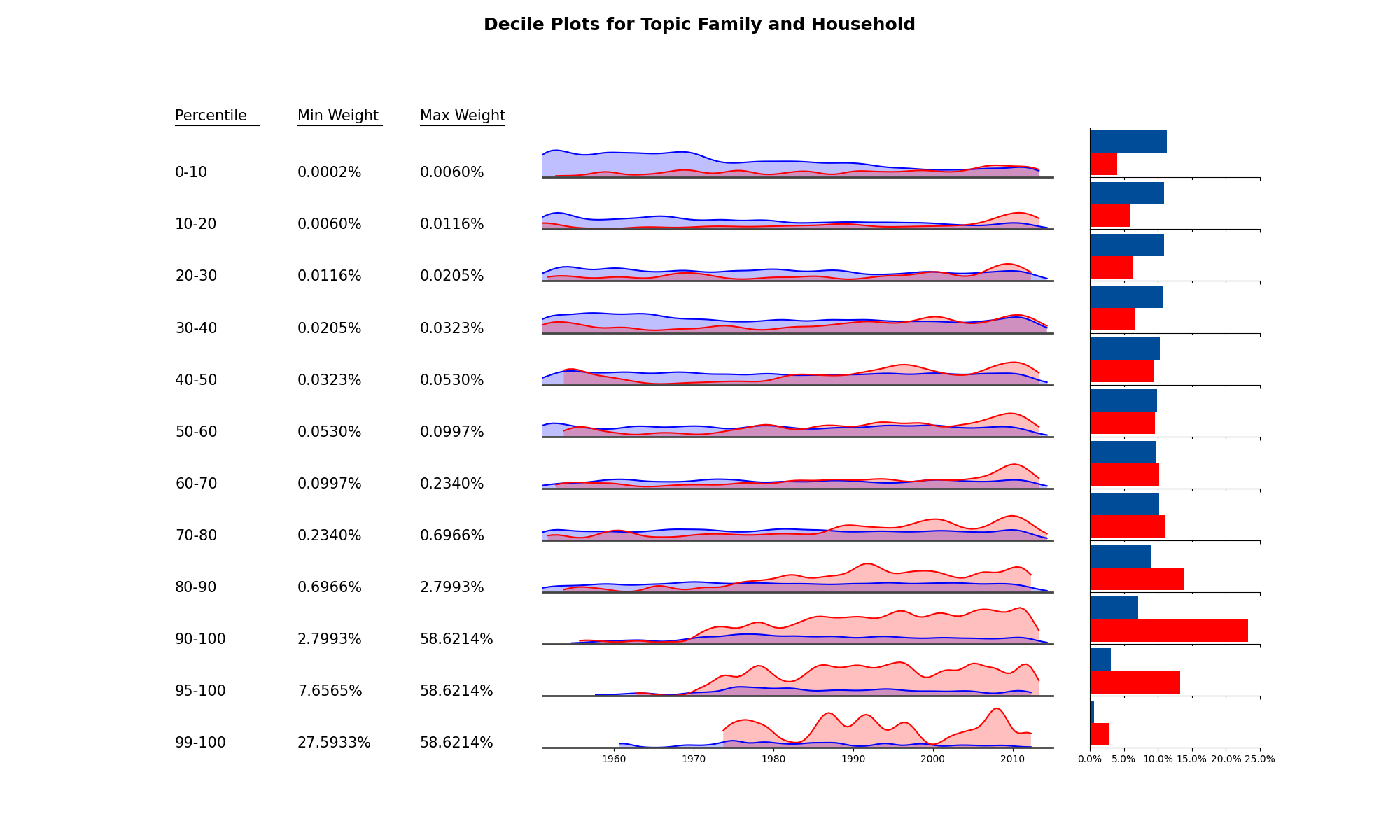

Supplement: S5 Fig — (TIF) [file pone.0262027.s007.tif]

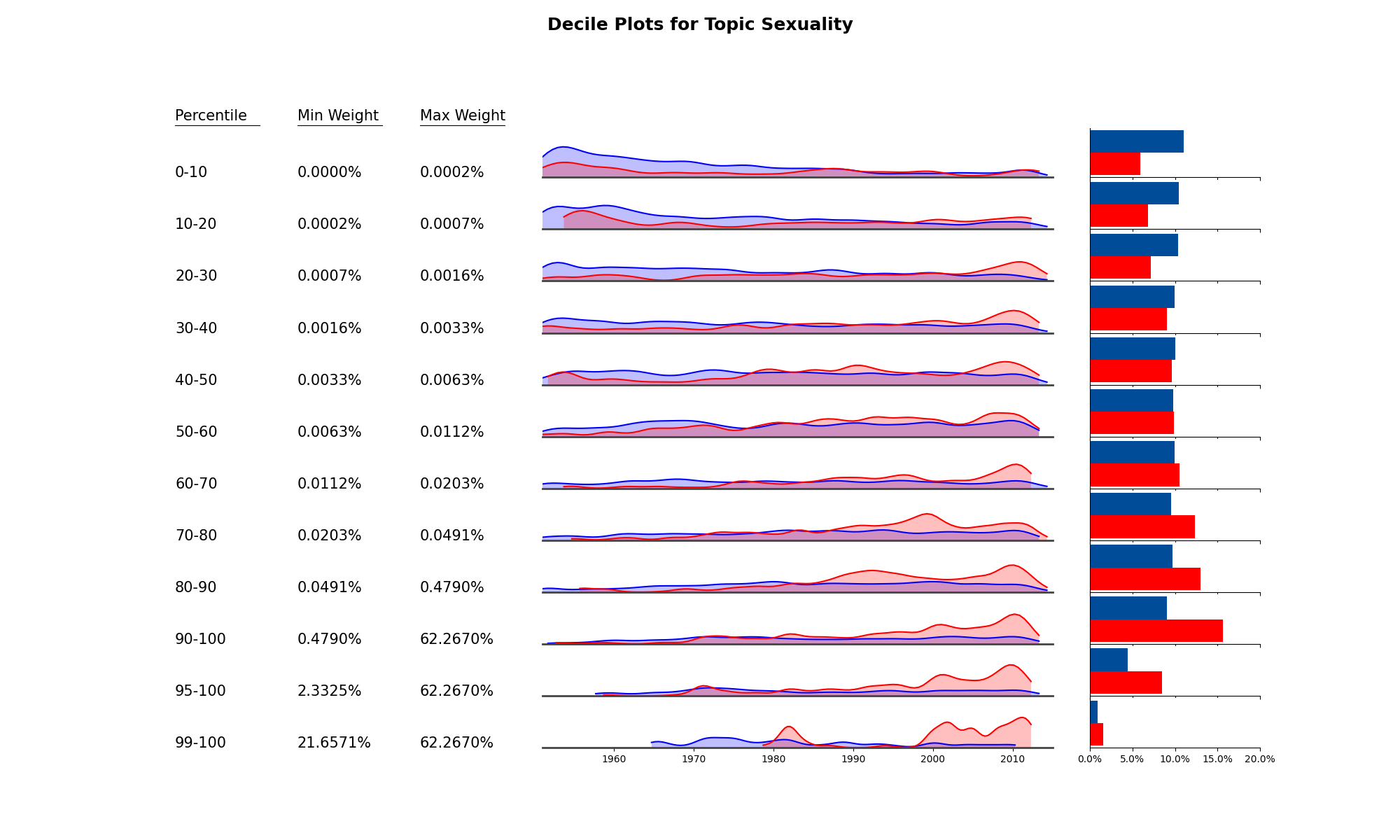

Supplement: S6 Fig — (TIF) [file pone.0262027.s008.tif]

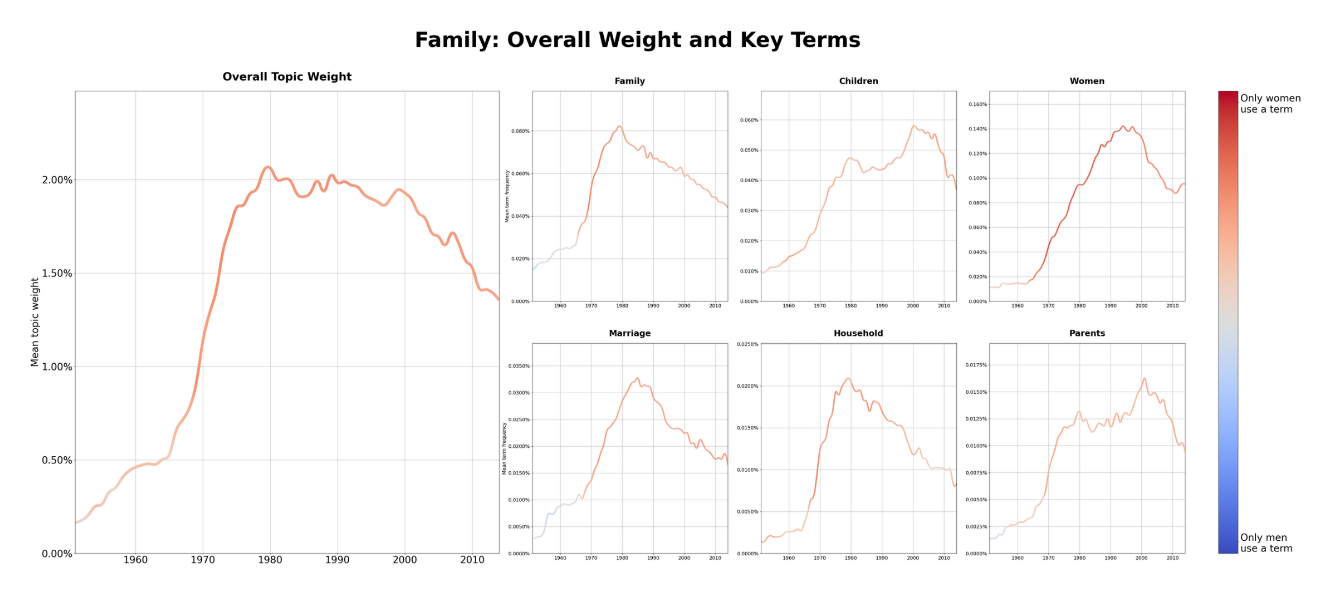

Supplement: S7 Fig — reports historical trends in the overall topic weight (large panel) and key terms related to the “family and household” topic (smaller panels). The trend-line color in the large panel indicates the gender composition of authors focusing on the “family and household” topic at a given point in time—red signals that women are dominating the topic, blue signals that men are dominating the topic. The trend-line color in the smaller panels specify developments in the gender composition of the authors using key terms (family, children, women, marriage, household, parents) within the “family and household” topic at a given point in time. The trend-line color was determined based on the following formula: avg_topic_weight(women) / (avg_topic_weight(women) + avg_topic_weight(men)). The topic graph shows the average weight across all articles, while the term charts show the average term frequency across all charts. The numbers in Fig 7 include co-authored articles that were written by men only (50 articles, 4.3% of the dataset) or women only (53 articles, 0.5% of the dataset). However, it excludes co-authored articles written by a mix of men and women (302 articles, 2.9% of the dataset). Women also write fewer journal articles and books than they do dissertations (20). (TIF) [file pone.0262027.s009.tif]

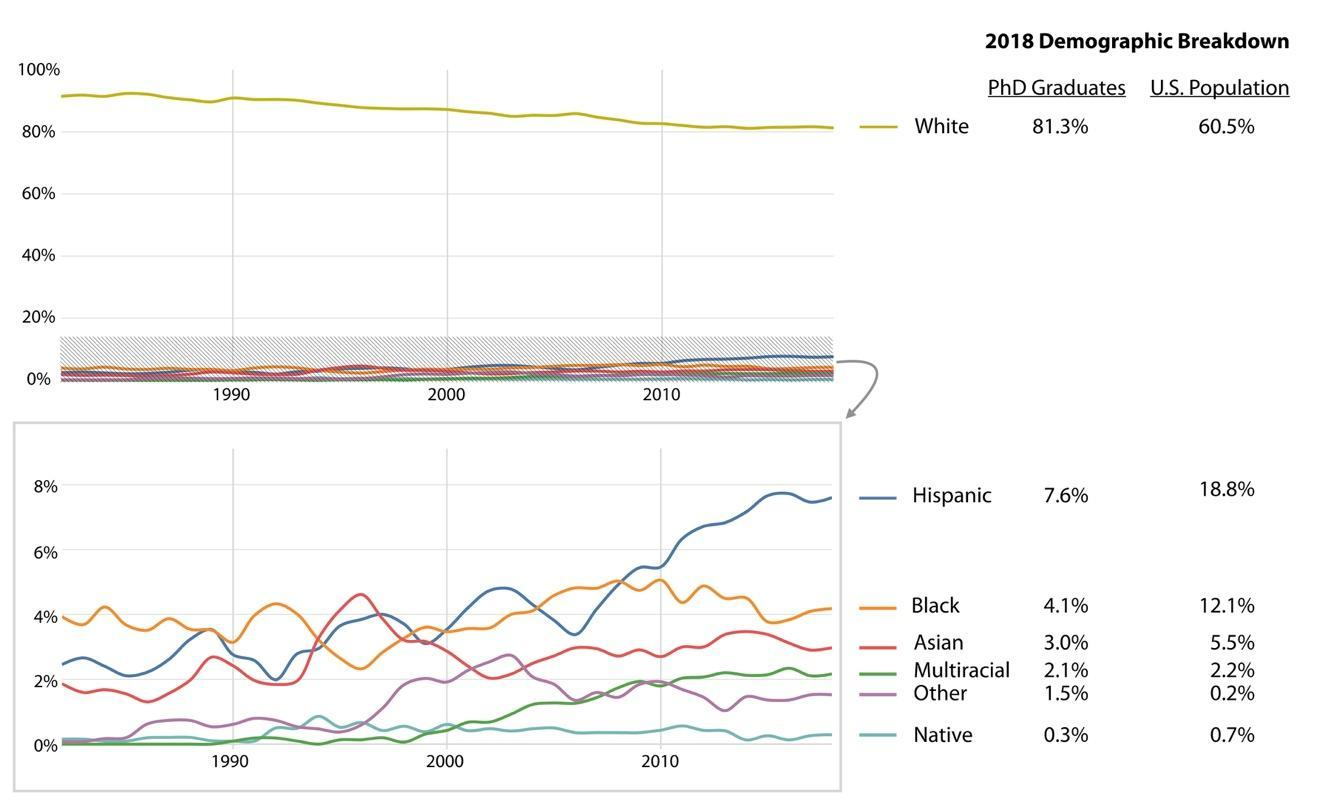

Supplement: S8 Fig — (TIF) [file pone.0262027.s010.tif]
